# Forest Orchids under Future Climate Scenarios: Habitat Suitability Modelling to Inform Conservation Strategies

**DOI:** 10.3390/plants13131810

**Published:** 2024-06-30

**Authors:** Antonio Pica, Daniele Vela, Sara Magrini

**Affiliations:** Department of Ecological and Biological Sciences, University of Tuscia, 01100 Viterbo, Italy; daniele.vela@unitus.it (D.V.); magrini@unitus.it (S.M.)

**Keywords:** biodiversity, protected areas (PAs), climate change, landscape ecology, *Cephalanthera*, *Epipactis*, *Limodorum*

## Abstract

*Orchidaceae* is one of the largest and most diverse families of flowering plants in the world but also one of the most threatened. Climate change is a global driver of plant distribution and may be the cause of their disappearance in some regions. Forest orchids are associated with specific biotic and abiotic environmental factors, that influence their local presence/absence. Changes in these conditions can lead to significant differences in species distribution. We studied three forest orchids belonging to different genera (*Cephalanthera*, *Epipactis* and *Limodorum*) for their potential current and future distribution in a protected area (PA) of the Northern Apennines. A Habitat Suitability Model was constructed for each species based on presence-only data and the Maximum Entropy algorithm (MaxEnt) was used for the modelling. Climatic, edaphic, topographic, anthropogenic and land cover variables were used as environmental predictors and processed in the model. The aim is to identify the environmental factors that most influence the current species distribution and the areas that are likely to contain habitats suitable for providing refuge for forest orchids and ensuring their survival under future scenarios. This will allow PA authorities to decide whether to invest more resources in conserving areas that are potential refuges for threatened species.

## 1. Introduction

The *Orchidaceae* is one of the largest plant families in the world, with many accepted species second only to the *Asteraceae* [1,2]. They are found in a wide variety of environments across the globe [3,4,5]. The number of orchid species is estimated to range from approximately 25,000 to 33,000 [2,6,7,8,9], belonging to 704–850 genera, according to various sources [6,9,10]. Furthermore, new orchid discoveries worldwide continue to increase these numbers [11,12,13,14]. In Europe, the number of species ranges from approximately 163 [3] to around 645 [15]. The most recent checklist of native flora indicates that there are 237 taxa of *Orchidaceae* in Italy [16,17], while other authors report 267 taxa [18].

The *Orchidaceae* family is widely recognised as having a significant number of endangered species, particularly among the terrestrial orchids [19,20,21]. Human activities, either by altering their natural habitats or by directly harming individual populations, are a major threat to these species [22,23,24]. Moreover, it has been observed that climate change can have a significant impact on the presence and distribution of certain species in a given territory [25,26,27]. As a result, the conservation of these species and their habitats has become a global priority to counteract their continued decline [28,29,30]. Orchids are widely recognised as umbrella and flagship species that can guide the conservation process to protect not only themselves but also other species growing in their habitats [29,31,32,33,34,35].

Habitat suitability models (HSMs) are broadly used to make prediction about potential distribution of habitats capable of hosting species at different scales, ranging from global [36,37] to local [38,39]. One of the most commonly used modelling methods for investigating the distribution of plant and animal species is the maximum entropy (MaxEnt) algorithm [40]. It is based on presence-only data and is known for its efficiency and also for turning out robust results, even when occurrence data is limited [41,42,43,44,45,46]. Species distribution models are frequently used to better conserve species, identify priority protection areas and plan effective interventions [47,48,49,50,51,52].

The disappearance of terrestrial orchids from temperate climates is mainly caused by the loss and deterioration of natural habitats [53,54]. However, human actions can help maintain past environmental conditions and ensure the presence of these species [4,55,56,57]. Furthermore, climate change may affect temperate terrestrial orchids in different ways. Depending on the species and region, they may respond positively or negatively to changes in climate parameters [58,59,60,61]. Consequently, further investigation is required to identify the impact of the aforementioned factors on these species.

The target species of this study are *Cephalanthera rubra* (L.) Rich., *Epipactis microphylla* (Ehrh.) Sw. and *Limodorum abortivum* (L.) Sw. These are three temperate terrestrial orchids whose native range is centred in Europe [10] and they belong to a widespread group of species known as forest orchids, which are mainly found in forest habitats. Forest orchids have a close relationship with the forest ecosystem as they can take up carbon from surrounding trees through mutualistic associations between higher plants and fungi, called mycorrhizae. These associations may or may not be obligatory to ensure their life cycle [62,63,64] and in the case of the species under investigation they are intimately associated with fungi throughout their life stages [63,65,66]. The target species exhibit a discontinuous presence with considerable gaps in the study area [67]. It is crucial to underscore that these species are linked to forest habitats, which are included in the Habitats Directive 92/43/EEC and are classified as priority (Natura 2000 code 9180*, 9210*, 9220*) or have a limited distribution within the PA (Natura 2000 code 9110, 9130, 91M0) [68]. Furthermore, the number of available occurrence points was considered sufficient to model species with the HSM approach [69].

Previous studies have extensively explored temperate European orchids, analysing the biotic and abiotic factors that influence their presence, abundance and distribution [4,55,70,71,72], including species commonly grown in forest environments [73,74,75]. However, it is important to note that only a limited number of studies have been conducted to assess the potential future distribution of a few species in relation to climate change using a species distribution modelling approach [25,76,77,78,79].

The objectives of this study were: (a) to investigate the potential present distribution of three forest orchid species in a protected area (PA) of Northern Apennines (Foreste Casentinesi National Park) and to predict the future distribution of habitats suitable for hosting them using the Habitat Suitability Model (MaxEnt); (b) to evaluate the impact of individual environmental factors on the species; (c) to assess the areas that are most suitable for the species in relation to future projections of global climate change and actual environmental conditions; (d) to provide the necessary guidance for future management and conservation projects by assessing in advance the most suitable areas for the conservation of forest orchids according to the model predictions. The aim is to provide a common methodological approach to help PAs manage and conserve threatened species for the long term while making efficient use of available resources.

## 2. Results

### 2.1. Species Occurrence and Environmental Variables

**Figure 1:**
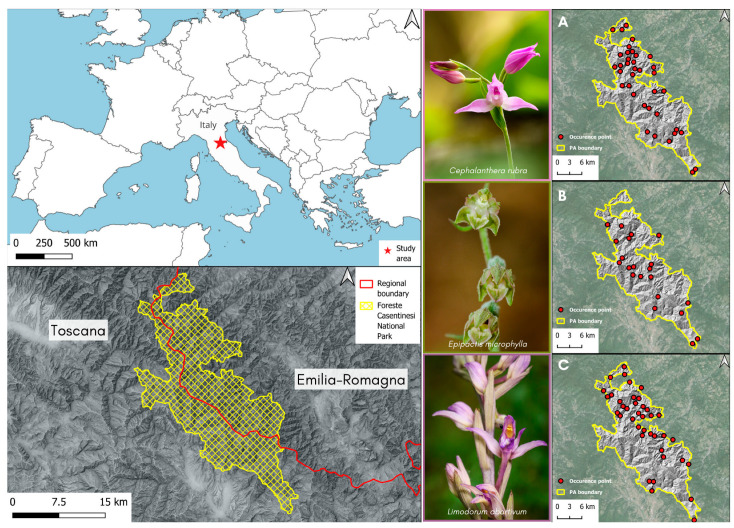
Study area (Foreste Casentinesi National Park, Northern Apennines) and reduced occurrence points of (**A**) *Cephalanthera rubra*, (**B**) *Epipactis microphylla* and (**C**) *Limodorum abortivum*.

A total of 149 occurrence records were used in this study to identify target species. These included *C. rubra* (54), *E. microphylla* (37), and *L. abortivum* (58). Following the thinning process, the number of occurrence points was reduced by 37, 21, and 39, respectively, in comparison to the original datasets (Figure 1, Table S1). 

Following the application of Pearson’s test and Variance Inflation Factor (VIF) (Table S2), the initial 46 environmental variables (EVs) were reduced to 12 for *C. rubra*, 7 for *E. microphylla*, and 13 for *L. abortivum*. Only the first five variables with the highest contribution to the model were isolated and used for the final model for each species (Table 1).

### 2.2. Model Optimization and Evaluation Results

The standard values of the MaxEnt software (RM = 1 and FC = LQHPT, where RM = regularization multipliers, FC = feature combinations, L = linear, Q = quadratic, H = hinge, P = product and T = threshold) employed in the initial screening phase of the model were modified to optimise the prediction results. The RM parameter was set to take values between 0.5 and 4, with increments of 0.5. This process yielded eight regularization multiplier values, which were associated with six combinations of one or more feature classes (L, LQ, H, LQH, LQHP, LQHPT). This resulted in a total of 48 parametric combinations that the model can take instead of the default parameters to test complexity. The optimal set of parameters was identified by selecting combinations with Akaike information criterion correction delta (ΔAICc) = 0. For *C. rubra*, the optimal parameters were FC = LQHPT and RM = 2.5; for *E. microphylla*, the optimal parameters were FC = L and RM = 0.5; for *L. abortivum*, the optimal parameters were FC = LQHP and RM = 2. The final distribution model built for *C. rubra* with its optimised parameters presents the values of Area Under Curve difference (AUC _DIFF_) = 0.05 ± 0.06 and 10% training omission rate (OR _10_) = 0.06; the final distribution model built for *E. microphylla* with its optimised parameters presents the values of AUC _DIFF_ = 0.05 ± 0.04 and OR _10_ = 0.06; the final distribution model built for *L. abortivum* with its optimised parameters presents the values of AUC _DIFF_ = 0.02 ± 0.03 and OR _10_ = 0.09. The accuracy and performance of the model were evaluated using the AUC training value (Figures S7–S9). For *C. rubra*, the value of AUC mean = 0.88 ± 0.03; for *E. microphylla*, the value of AUC mean = 0.91 ± 0.02; for *L. abortivum*, the value of AUC mean = 0.86 ± 0.02.

The Maximum Test Sensitivity Plus Specificity (MTSPS) value obtained from the final MaxEnt model for *C. rubra* was 0.4062. The probability (*p*) obtained from the model was used to classify the output as follows: unsuitability (*p* < 0.4062), low suitability (0.4062 < *p* < 0.6041), moderate suitability (0.6041 < *p* < 0.8021) and high suitability (*p* > 0.8021). The MTSPS value obtained from the final MaxEnt model for *E. microphylla* was 0.2683. The probability (*p*) obtained from the model was used to classify the output as follows: unsuitability (*p* < 0.2683), low suitability (0.2683 < *p* < 0.5122), moderate suitability (0.5122 < *p* < 0.7561) and high suitability (*p* > 0.7561). The MTSPS value obtained from the final MaxEnt model for *L. abortivum* was 0.6144. The probability (p) obtained from the model was used to classify the output as follows: unsuitability (*p* < 0.6144), low suitability (0.6144 < *p* < 0.7429), moderate suitability (0.7429 < *p* < 0.8715) and high suitability (*p* > 0.8715).

### 2.3. Contribution of Environmental Variables

**Figure 2:**
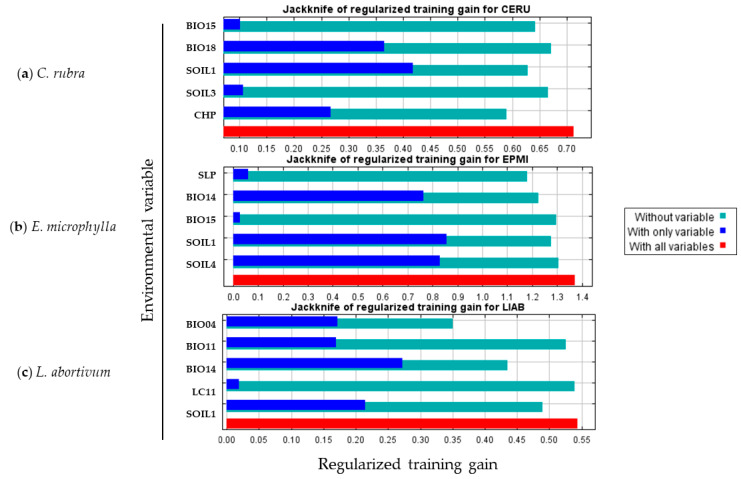
Jackknife of regularized training gain test for *C. rubra* (**a**), *E. microphylla* (**b**) and *L. abortivum* (**c**).

The environmental variables influencing the distribution of each species are listed below, derived from the jackknife test applied to them (Figure 2). The higher the contribution, the greater the influence of that variable in the final prediction. 

In *C. rubra*, the variables most influencing the distribution in the area, in terms of permutation importance, are the bulk density of the fine earth fraction (SOIL1), the precipitation of the warmest quarter (BIO18), the precipitation seasonality (BIO15), the cumulative human pressure on the environment (CHP) and the volumetric fraction of coarse fragments (SOIL3). Consequently, the variables in question exhibit a corresponding permutation importance in the distribution prediction of 35.5%, 25.2%, 18.5%, 11.4% and 9.5%. In terms of percent contribution to the model, the values are 34%, 24.7%, 8.1%, 27.4% and 5.8%, respectively (Table 2). In *E. microphylla*, the variables most influential in determining distribution in the area, as determined by permutation importance, are the bulk density of the fine earth fraction (SOIL1), the precipitation of the driest month (BIO14), the proportion of clay particles in the fine earth fraction (SOIL4), the slope (SLOPE) and the precipitation seasonality (BIO15). Consequently, the permutation importance of each variable is as follows: 46.6%, 19%, 17.6%, 12.5% and 10.4%. In terms of percent contribution to the model, the values are 45%, 18.3%, 21.4%, 8.6% and 6.7%, respectively (Table 2). Finally, in *L. abortivum*, the variables most influencing the distribution, according to permutation importance, are the temperature seasonality (BIO04), the precipitation of the driest month (BIO14), the bulk density of the fine earth fraction (SOIL1), the mean temperature of the coldest quarter (BIO11) and the barrenness (LC11). Consequently, the permutation importance of each variable is as follows: 39.5%, 35.2%, 17.7%, 6.7% and 1%. In terms of percent contribution to the model, the values are 30.2%, 32.5%, 28.9%, 7.4% and 1%, respectively (Table 2). 

The five most influential variables in the final model for each species are presented below. For each variable, the range of suitable values for the presence of the investigated species was indicated, that is, the values associated with all areas classified as suitable for the species. In addition, the range of optimum values for the species was indicated, that is, only the values associated with the areas classified as high suitable. Furthermore, response curves for each environmental variable included in the final model were generated (Figures S4–S6). 

In *C. rubra*, the precipitation seasonality (BIO15) value range was 23.64–32.56% and its optimum was 26.26–32.01%; precipitation of warmest quarter (BIO18) range was 222–273 mm and its optimum was 232–268 mm; bulk density of the fine earth fraction (SOIL1) range was 83.30–102.81 cg/cm^3^ and its optimum was 83.30–96.96 cg/cm^3^; volumetric fraction of coarse fragments (SOIL3) range was 99.46–153.97 cm^3^/dm^3^ (vol‰) and its optimum was 111.37–148.68 cm^3^/dm^3^ (vol‰); cumulative human pressure on the environment (CHP) range was 3.00–18.75 and its optimum was 3.00–6.25.

In *E. microphylla*, the precipitation of driest month (BIO14) value range was 60–69 mm and its optimum was 63–69 mm; precipitation seasonality (BIO15) value range was 23.64–32.59% and its optimum was 23.64–31.44%; bulk density of the fine earth fraction (SOIL1) range was 83.30–98.33 cg/cm^3^ and its optimum was 83.30–91.43 cg/cm^3^; the proportion of clay particles within the fine earth fraction (SOIL4) value range was 188.77–317.77 g/kg and its optimum was 188.77–244.62 g/kg; slope (SLP) value range was 0.66–16.06 degree and its optimum was 1.43–10.03 degree. 

In *L. abortivum*, the temperature seasonality (BIO04) value range was 637.23–665.20% and its optimum was 645.61–661.48%; mean temperature of coldest quarter (BIO11) value range was 1.58–3.77 °C and its optimum was 1.95–3.65 °C; precipitation of driest month (BIO14) value range was 60–68 mm and its optimum was 62–67 mm; land cover class barren (LC11) value range was 0–34% and its optimum was 0–12%; bulk density of the fine earth fraction (SOIL1) range was 83.68–102.81 cg/cm^3^ and its optimum was 83.68–95.07 cg/cm^3^.

### 2.4. Prediction of Potential Suitable Habitat Distribution under Current Climate Conditions

**Figure 3:**
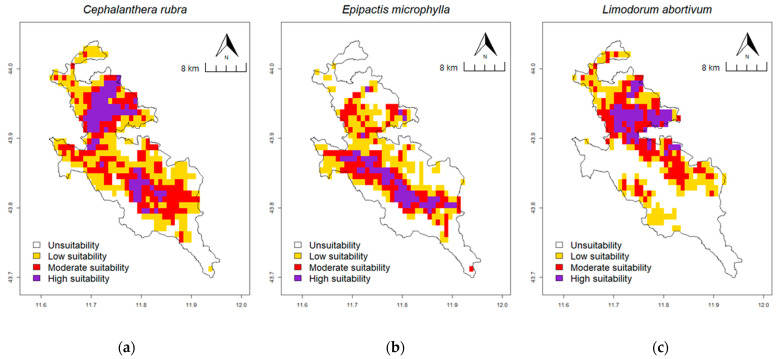
Prediction of potential suitable habitats distribution under current climate conditions elaborated by MaxEnt model: *C. rubra* (**a**), *E. microphylla* (**b**) and *L. abortivum* (**c**).

The calibrated and optimised MaxEnt prediction model was employed to predict the local distribution of habitats suitable for the presence of the investigated species under current climatic conditions (Figure 3). In *C. rubra* (Figure 3a), the estimated area occupied under current conditions by potentially suitable habitats is 315.28 km^2^, which represents 76.3% of the total area analysed. With regard to the suitability of habitats for the species, the high suitability area covers 56.94 km^2^, the moderate suitability area covers 113.89 km^2^, and the low suitability area covers 144.44 km^2^, respectively representing 13.78%, 27.56% and 34.96% of the total area. Finally, the area with habitats that are potentially unsuitable for the species is 97.92 km^2^ (23.70% of the total) (Table 3). In *E. microphylla* (Figure 3b), the estimated area occupied under current conditions by potentially suitable habitats is 225.69 km^2^ or 54.62% of the total area analysed. In terms of habitat suitability classes that could potentially host the species, the high suitability area covers 38.89 km^2^, the moderate suitability area covers 79.17 km^2^, and the low suitability area covers 107.64 km^2^, respectively representing 9.41%, 19.16% and 26.05% of the total area. Finally, the area with potentially unsuitable habitats for the species is 187.50 km^2^ (45.38% of the total) (Table 4). In *L. abortivum* (Figure 3c), the estimated area occupied under current conditions by potentially suitable habitats is 217.36 km^2^ or 52.61% of the total area analysed. About the habitat suitability classes for the species, the high suitability area covers 40.97 km^2^, the moderate suitability area covers 82.64 km^2^, and the low suitability area covers 93.75 km^2^, respectively representing 9.92%, 20% and 22.69% of the total. Lastly, the area with potentially unsuitable habitats for the species is 195.83 km^2^ (47.39% of the total) (Table 5).

### 2.5. Prediction of Potential Suitable Habitat Distribution under Future Climate Conditions

The potential distribution of suitable habitats for hosting the three investigated species was estimated for two different scenarios (Shared Socioeconomic Pathways), SSP2 4.5 and SSP5 8.5 (referred below as SSP245 and SSP585) and for two future year intervals, i.e., 2021–2040 and 2041–2060, which are referred to as the 2030s and 2050s, respectively. 

**Figure 4:**
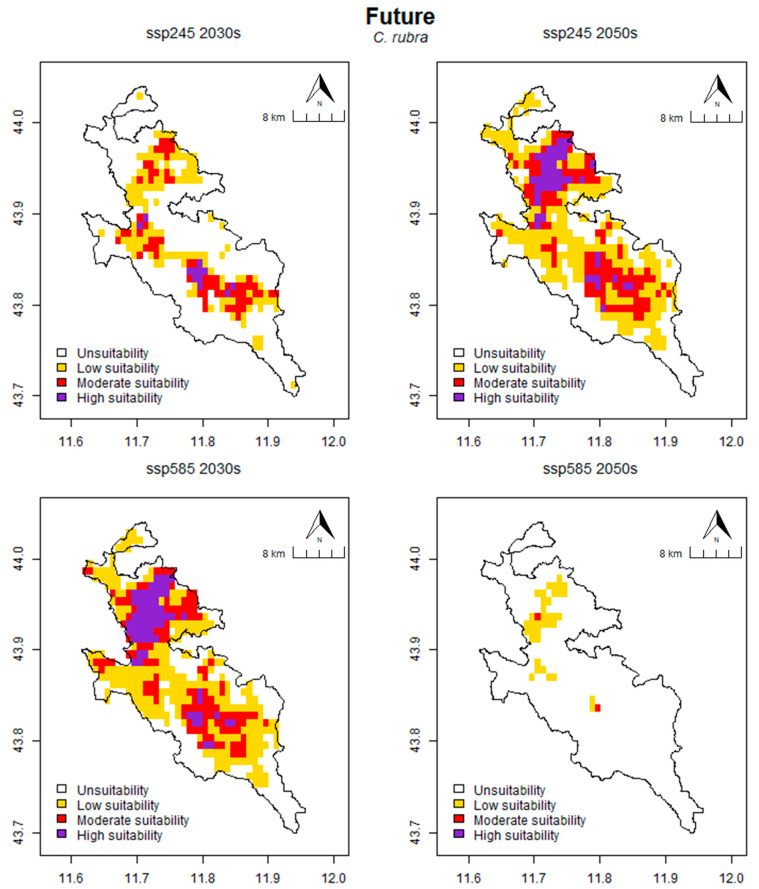
Prediction of potential suitable habitat distribution of *C. rubra* under future climate conditions.

In *C. rubra* (Figure 4), the predicted area of habitats suitable for the species under the SSP245 scenario covers 140.28 km^2^ for the 2030s and 281.94 km^2^ for the 2050s, representing 33.95% and 68.24% of the total area, respectively. In the SSP585 scenario, the area of suitable habitat is 294.44 km^2^ for the 2030s and 27.08 km^2^ for the 2050s, representing 71.26% and 6.55% of the total area, respectively. A review of the habitat suitability classes for the SSP245 scenario reveals that the area is classified as follows: for the 2030s, 272.92 km^2^ (66.05%) is unsuitable, 85.42 km^2^ (20.67%) is low suitable, 46.53 km^2^ (11. 26%) is moderate suitable and 8.33 km^2^ (2.02%) is high suitable; for the 2050s, 131.25 km^2^ (31.76%) is unsuitable, 166.67 km^2^ (40.34%) is low suitable, 80.56 km^2^ (19.50%) is moderate suitable and 34.72 km^2^ (8.40%) is high suitable. For the SSP585 scenario area is classified as follows: for the 2030s, 118.75 km^2^ (28.74%) is unsuitable, 166.67 km^2^ (40.34%) is low suitable, 81.25 km^2^ (19.66%) is moderately suitable and 46.53 km^2^ (11. 26%) is high suitable; for 2050s, 386.11 km^2^ (93.45%) is unsuitable, 25.69 km^2^ (6.22%) is low suitable, 1.39 km^2^ (0.34%) is moderate suitable and no area is classified as high suitable (Table 3).

**Figure 5:**
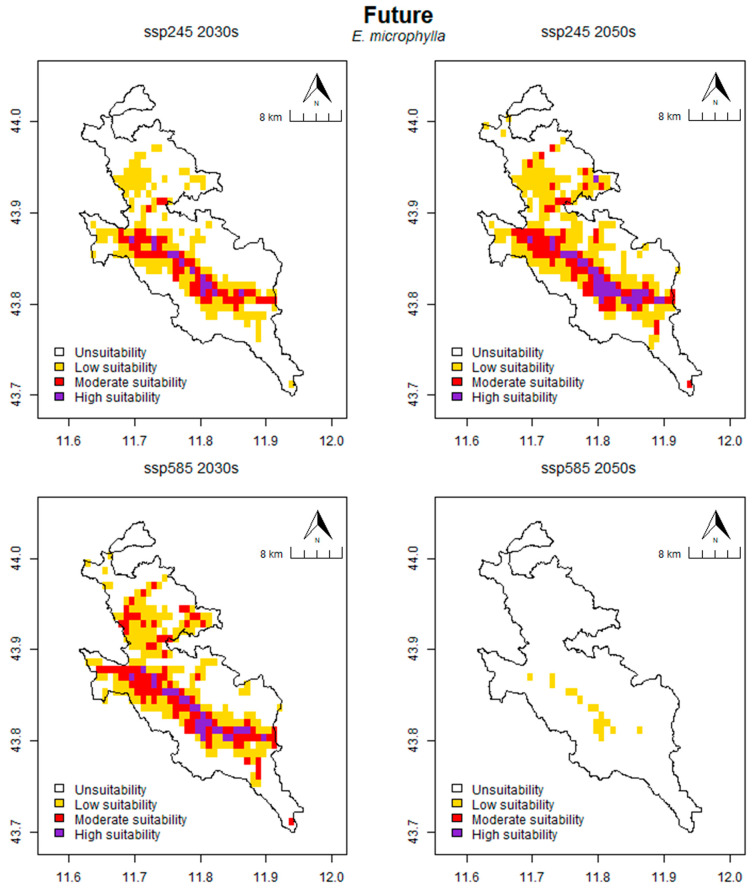
Prediction of potential suitable habitat distribution of *E. microphylla* under future climate conditions.

In *E. microphylla* (Figure 5), the prediction of suitable habitats for the species covers an area of 144.44 km^2^ for the SSP245 scenario in the 2030s and 195.14 km^2^ for the 2050s, respectively representing 34.96% and 47.23% of the total area. For the SSP585 scenario, the suitable area is 198.61 km^2^ for the 2030s and 11.81 km^2^ for the 2050s, representing 48.07% and 2.86% of the total area, respectively. Further analysis of the habitat suitability classes for the SSP245 scenario reveals that the area is distributed as follows: for the 2030s, 268.75 km^2^ (65.04%) is unsuitable, 97.92 km^2^ (23.70%) is low suitable, 37.50 km^2^ (9.08%) is moderately suitable and 9.03 km^2^ (2.18%) is high suitable. The area is distributed as follows for the 2050s: 218.06 km^2^ (52.77%) is unsuitable, 120.83 km^2^ (29.24%) is low suitable, 51.39 km^2^ (12.44%) is moderately suitable and 22.92 km^2^ (5.55%) is high suitable. With regard to the SSP585 scenario, the surface area is distributed as follows: for the 2030s, 214.58 km^2^ (51.93%) is unsuitable, 115.97 km^2^ (28.07%) is low suitable, 64.58 km^2^ (15.63%) is moderately suitable and 18.06 km^2^ (4.37%) is high suitable. Finally, the area is distributed as follows for the 2050s: 401.39 km^2^ (97.14%), is designated as unsuitable, 11.81 km^2^ (2.86%) is classified as low suitable and no area is classified as moderate suitable or high suitable (Table 4).

**Figure 6:**
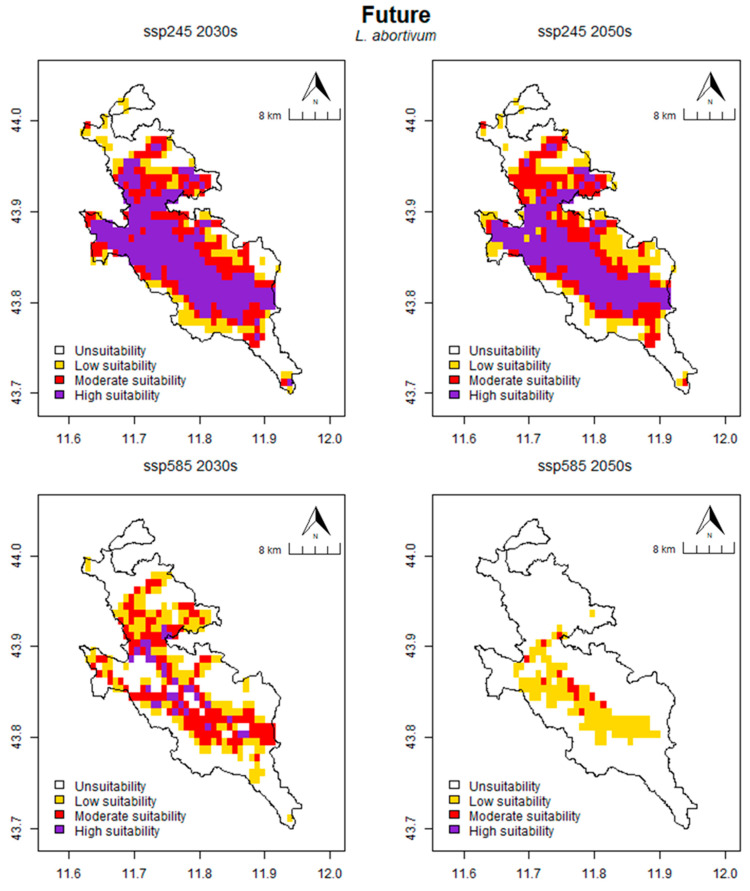
Prediction of potential suitable habitat distribution of *L. abortivum* under future climate conditions.

In *L. abortivum* (Figure 6), the projected suitable habitat area for the species under the SSP245 scenario is 320.83 km^2^ for the 2030s and 309.03 km^2^ for the 2050s. This represents 77.65% and 74.79% of the total area, respectively. For the SSP585 scenario, the eligible area is 202.78 km^2^ for the 2030s and 79.17 km^2^ for the 2050s, respectively, representing 49.08% and 19.16% of the total area. Further analysis of the various habitat suitability classes for the SSP245 scenario reveals that the area is distributed as follows: for the 2030s, 92.36 km^2^ (22.35%) is unsuitable, 59.72 km^2^ (14.45%) is low suitable, 95.14 km^2^ (23.03%) is moderately suitable and 165.97 km^2^ (40.17%) is high suitable. For the 2050s, 104.17 km^2^ (25.21%) is unsuitable, 75.69 km^2^ (18.32%) is low suitable, 100.69 km^2^ (24.37%) is moderately suitable and 132.64 km^2^ (32.10%) is high suitable. The distribution of the area for the SSP585 scenario is as follows: for the 2030s, 210.42 km^2^ (50.92%) is unsuitable, 91.67 km^2^ (22.18%) is low suitable, 90.97 km^2^ (22.02%) is moderately suitable and 20.14 km^2^ (4.87%) is of high suitable. Finally, for the 2050s, 334.03 km^2^ (80.84%) is unsuitable, 70.83 km^2^ (17.14%) is low suitable, 8.33 km^2^ (2.02%) is moderately suitable and no area is classified as highly suitable (Table 5).

### 2.6. Distribution Changes under Future Climate Conditions

The future distribution range of each species is presented in three types of cell classes: loss, stable and gain. The loss class corresponds to the amount of cells predicted to be lost, the stable class corresponds to the amount of cells actually occupied and the gain class corresponds to the amount of cells predicted to be gained in relation to the present distribution of each species. In addition, the percentage of cells currently occupied and predicted to be lost (Loss/(Loss + Stable)) is referred to as %Loss, while the percentage of cells predicted to be gained in comparison to the number of cells currently occupied (Gain/(Loss + Stable)) is designated as %Gain. The Species Range Change (SRC) is defined as the percentage of cells predicted to change (loss or gain) in comparison to the number of cells currently occupied (%Gain—%Loss). Finally, the Current Range Size (CRS) is the number of cells currently occupied. 

**Figure 7:**
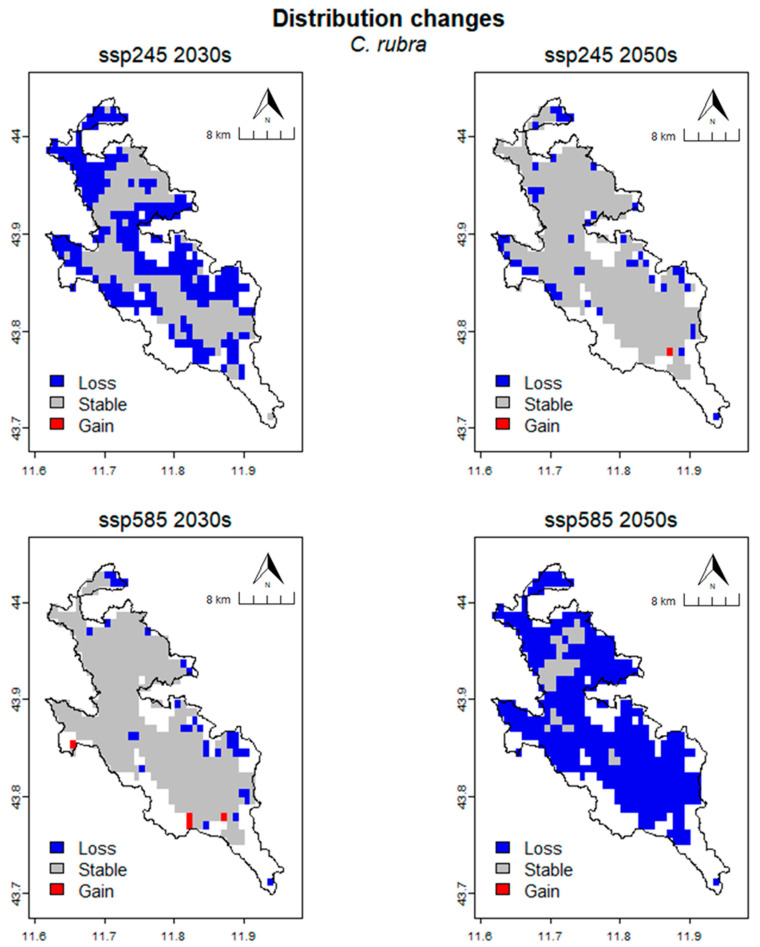
Spatial changing in habitat suitabilty for different climate scenarios in *C. rubra*.

In *C. rubra* (Figure 7), the CRS is 315.28 km^2^ (454 cells). For the SSP245 scenario, in the 2030s, the area predicted to be lost is 175.00 km^2^ (252 cells), the area predicted to be stable is 140.28 km^2^ (202 cells), and no cells are predicted to be gained from the CRS. The percentage loss (%Loss), percentage gain (%Gain) and SRC are 55.51%, 0% and −55.51% respectively. For the 2050s, the occupied area predicted to be lost is 34.03 km^2^ (49 cells), the stable area is 281.25 km^2^ (405 cells) and the gained area is 0.69 km^2^ (1 cell). The percentage loss, percentage gain and SRC are 10.79%, 0.22% and −10.57%, respectively. In the SSP585 scenario, the area predicted to be lost in the 2030s is 23.61 km^2^ (34 cells), the area predicted to be stable is 291.67 km^2^ (420 cells) and the area predicted to be gained is 2.78 km^2^ (4 cells). The percentage loss, percentage gain, and SRC are 7.49%, 0.88%, and −6.61%, respectively. For the 2050s, the occupied area predicted to be lost is 288.19 km^2^ (415 cells), the stable area is 27.08 km^2^ (39 cells), and no cells are gained compared to the CRS. The percentage loss, percentage gain, and SRC are 91.41%, 0%, and −91.41%, respectively (Table 6).

**Figure 8:**
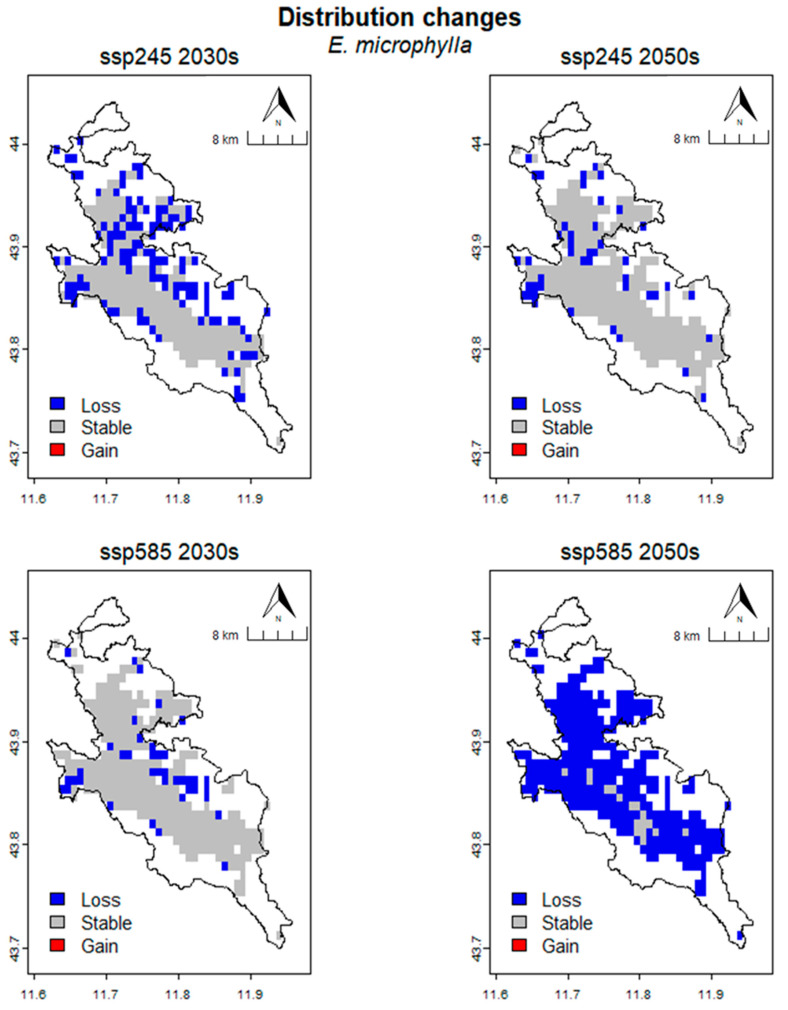
Spatial changing in habitat suitabilty for different climate scenarios in *E. microphylla*.

In *E. microphylla* (Figure 8), the CRS is 225.69 km^2^ (315 cells). For the SSP245 scenario, in the 2030s, the area predicted to be lost is 81.25 km^2^ (117 cells), the area predicted to be stable is 144.44 km^2^ (208 cells), and no cells are predicted to be gained from the CRS. The percentage loss (%Loss), percentage gain (%Gain) and SRC are 36.00%, 0% and −36.00% respectively. For the 2050s, the occupied area predicted to be lost is 30.56 km^2^ (44 cells), the stable area is 195.14 km^2^ (281 cells) and no cells were gained in comparison to the CRS. The percentage loss, percentage gain and SRC are 13.54%, 0% and −13.54% respectively. For the SSP585 scenario, for the 2030s, the occupied area predicted to be lost is 27.08 km^2^ (39 cells), and the stable area is 198.61 km^2^ (286 cells) while no cells are gained compared to the CRS. The percentage loss, percentage gain and SRC are 12.00%, 0% and −12.00% respectively. For the 2050s, the occupied area predicted to be lost is 213.89 km^2^ (308 cells), the stable area is 11.81 km^2^ (17 cells) and no cells were gained compared to the CRS. The percentage loss, percentage gain, and SRC are 94.77%, 0%, and −94.77%, respectively (Table 7).

**Figure 9:**
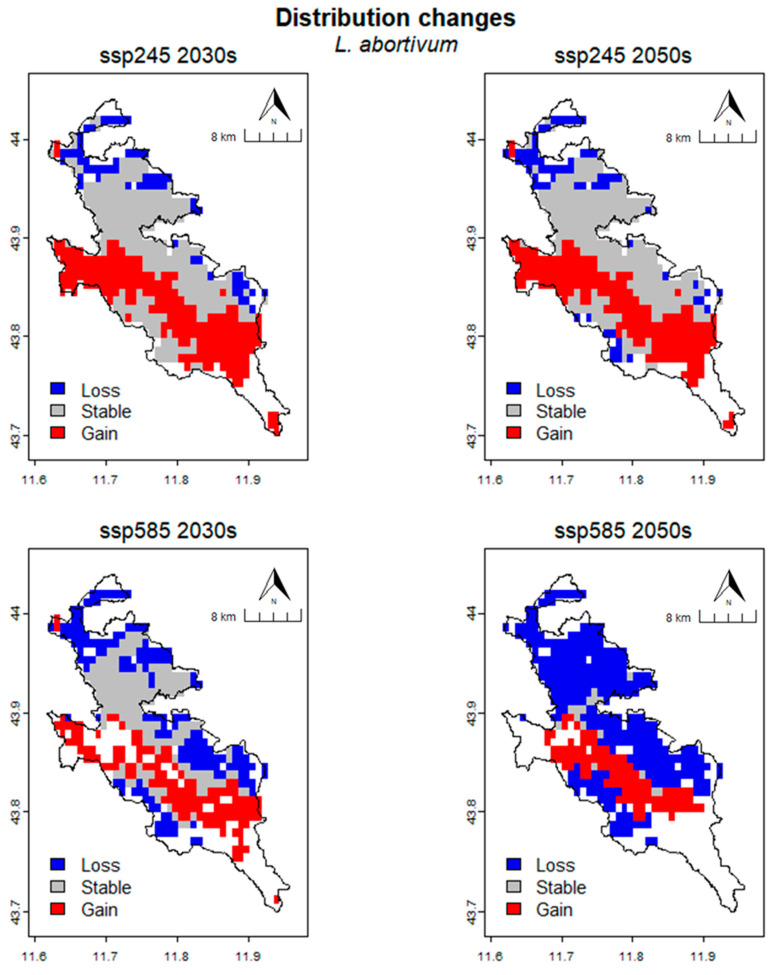
Spatial changing in habitat suitabilty for different climate scenarios in *L. abortivum*.

In *L. abortivum* (Figure 9), the CRS is 217.36 km^2^ (313 cells). For the SSP245 scenario, for the 2030s, the occupied area predicted to be lost is 41.67 km^2^ (60 cells), the stable area is 175.69 km^2^ (253 cells) and the gained area is 145.14 km^2^ (209 cells). The percentage loss (%Loss), percentage gain (%Gain) and SRC are 19.17%, 66.77% and 47.60% respectively. For the 2050s, the occupied area predicted to be lost is 44.44 km^2^ (64 cells), the stable area is 172.92 km^2^ (249 cells) and the gained area is 136.11 km^2^ (196 cells). The percentage loss, percentage gain and SRC are 20.45%, 62.62% and 42.17% respectively. For the SSP585 scenario, for the 2030s, the occupied area predicted to be lost is 105.56 km^2^ (152 cells), the stable area is 111.81 km^2^ (161 cells) and the gained area is 90.97 km^2^ (131 cells). The percentage loss, percentage gain and SRC are 48.56%, 41.85% and −6.71% respectively. For the 2050s, the occupied area predicted to be lost is 206.25 km^2^ (297 cells), the stable area is 11.11 km^2^ (16 cells) and the gained area is 68.06 km^2^ (98 cells). The percentage loss, percentage gain and SRC are 94.89%, 31.31% and −63.58% respectively (Table 8).

## 3. Discussion

The model performance and accuracy evaluation yielded satisfactory results. The AUC _DIFF_ and OR _10_ values for the three species were below the threshold values indicated by other studies [80,81]. This indicates that the models developed are not subject to overfitting. The model’s accuracy, as assessed by the AUC value of the ROC (Figures S7–S9), can be considered good for *C. rubra* (0.88 ± 0.03) and *L. abortivum* (0.86 ± 0.02) and excellent for *E. microphylla* (0.91 ± 0.02) [82,83,84]. When considering the distribution of the three potential suitable habitats under current climate conditions, it can be observed that the largest potential suitable area in terms of extent is that of *C. rubra* (315.28 km^2^), followed by *E. microphylla* (225.69 km^2^) and *L. abortivum* (217.36 km^2^), which have similar values. Furthermore, when considering each of the three suitability classes, *C. rubra* exhibits the highest area values. In contrast, *E. microphylla* exhibits higher values of suitable surface area exclusively within the low suitability class, in comparison to *L. abortivum*. The current potential distribution of the three species exhibits distinct patterns. In *E. microphylla*, the highly suitable area is concentrated on the Apennine ridge line, specifically at the higher altitudes of the PA. Conversely, in *L. abortivum*, the areas of highest suitability are concentrated in the lower altitudes of the northern and north-eastern portion of the PA, with some disjunct occupied cells (low suitability and moderate suitability) in the south-western slope. The areas suitable for the presence of *C. rubra* exhibit a more homogeneous distribution throughout the PA, with two distinct high-suitability cores. The largest of these is located in the north-northeast portion of the PA, while the other is situated in the central area of the PA at higher altitudes.

The analysis of the individual variables influencing the distribution of the three species in the area under consideration revealed a greater involvement of the variables associated with precipitation (BIO04, BIO11, BIO14, BIO15, BIO18) and the variables associated with the soil (SOIL1, SOIL3, SOIL4). The bulk density of the fine earth fraction (SOIL1) was found to be present in all three species, influencing their distribution. The jackknife test in *C. rubra* and *E. microphylla* shows that this variable contributes most to the model when used in isolation (dark blue bar). The values of this variable range from a minimum of 83.30 cg/cm^3^ to a maximum of 102.81 cg/cm^3^. The optimal values for each species are situated within this range and exhibit minimal variation, with *C. rubra* exhibiting the highest optimal value (96.96 cg/cm^3^) in comparison to the other two species. The bulk density of the soil is dependent upon the presence of soil pores and soil moisture, both of which are fundamental for the presence of orchids [4]. Precipitation seasonality (BIO15) represents one of the climatic variables influencing the distribution of habitats suitable for *C. rubra* and *E. microphylla*. This variable is frequently correlated with the distribution of orchids [85] and can affect their presence in different ways by acting as a limiting factor to their survival. For example, it has been demonstrated that summer rainfall in rare forest species at the southern limit of their distribution [86] and the slope of the terrain [87] can both exert a significant influence on the distribution of these species. The two species exhibit partial ecological requirements in the study area. The jackknife test with the variable alone indicates that it makes the least contribution to the models of the two species. In *C. rubra*, the precipitation of the warmest quarter (BIO18) value has a strong influence on the distribution. The response curve explains how low precipitation values in the warmer months proportionally limit the probability of suitable habitats. This variable also correlates with the continental distribution of orchids [85], thereby reinforcing the importance of precipitation as a limiting factor [86]. Furthermore, the volumetric fraction of coarse fragments (SOIL3) and the cumulative human pressure on the environment (CHP) also exert a partial influence on the distribution. In the former variable, there is an increase in probability that is proportional to the increase in the fraction of coarse fragments; in the latter, the probability decreases rapidly with increasing human pressure. In *E. microphylla*, the precipitation of the driest month (BIO14) and the proportion of clay particles (0.002 mm) within the fine earth fraction (SOIL4) were found to be very influential factors in determining the distribution. The precipitation of the driest month (BIO14) is an important climatic variable that appears to be related to the distribution of orchids [85]. According to Djordjević and Tsiftsis [4], soil texture can influence moisture content through its retaining capacity. However, there is a lack of studies on orchids [4]. The response curves (Figure S5) indicate that the limiting factor is the low precipitation values in the driest month, and, conversely, the high clay content in the fine earth fraction. The distribution of *L. abortivum* appears to be most effectively explained by temperature seasonality (BIO04) and precipitation of the driest month (BIO14). Temperature seasonality appears to be among the environmental variables most frequently associated with the spread of orchids across the globe [88,89]. Additionally, the mean temperature of the coldest quarter (BIO11) exerts an influence on the distribution of *L. abortivum*. Upon examination of the BIO11 response curve (Figure S6), it can be observed that an increase in the mean temperature value during winter is associated with a net decrease in the probability value of the presence of suitable habitats, acting as a limiting factor.

The results indicate that habitats with the potential to host the studied species under future climate conditions are likely to be significantly impacted. The scenario with the greatest loss of suitable habitat, in terms of area, is the SSP585 in the 2050s, with a loss of suitable area of 288.19 km^2^ in *C. rubra*, 213.89 km^2^ in *E. microphylla* and 206.25 km^2^ in *L. abortivum*. In all three cases, the percentage of lost area exceeded 90%. This trend has been observed worldwide and in Europe for both terrestrial and epiphytic orchids [90], as well as for other plant species [91], although in many cases it seems to have positive effects, leading to an expansion of the range suitable for the species [92,93]. Furthermore, there is a clear distinction in the potentially suitable area of *L. abortivum*, which is the only species in which the habitats suitable for it in that climatic situation appear to shift towards the ridge areas with higher altitudes. In particular, the 98 cells (68.06 km^2^) gained in comparison to the current distribution are located in the ridge areas. The upward altitudinal shift of the area potentially occupied by suitable habitats for *L. abortivum* is on average 331 m compared to the mean altitude occupied by suitable areas under current climatic conditions. In this case, the suitable areas will be distributed between 939 m and 1420 m a.s.l. Altitudinal shifting is a frequent dynamic in previous literature, and a considerable number of studies have documented altitudinal shifting of suitable habitats [94,95,96,97], with some shifting to higher altitudes [90] and others to lower altitudes than the original distribution [83]. A comparison of the values of area lost and gained reveals a similar dynamic for *C. rubra* and *E. microphylla*. The scenario with the least area lost is SSP585 2030s, with 7.49% and 12.00% of cells lost, respectively. In both cases, the percentage of area gained is below 1%. Similarly, for the SSP245 2050s scenario, the values of the area lost are comparable to those observed in the SSP585 2030s scenario, although slightly higher (10.79% and 13.54%). The intermediate values of the area lost in the SSP245 2030s scenario for *C. rubra* and *E. microphylla* are 55.51% and 36.00% respectively. In contrast, the SSP245 2030s scenario for *L. abortivum* represents the optimal outcome, with the highest value of suitable habitat area gained (145.14 km^2^) and the lowest loss of occupied area (41.67 km^2^). This increase for the SSP245 scenario has also been verified in other studies [83,98,99]. In this case, the Species Range Change (SRC) is the highest (47.60) compared to all other scenarios. The SSP585 2030s and SSP245 2050s scenarios for *L. abortivum* species present an intermediate situation. In the SSP245 2050s scenario, the area of suitable habitat lost and gained in comparison to the current conditions is comparable to that of the SSP245 2030s scenario, although with a higher percentage of area lost (20.45%) in contrast to a lower value of area gained (62.62%). In the SSP585 2030s scenario, the values of lost and gained area are almost equivalent, with a negative SRC value of −6.71. In consideration of the SSP245 scenario, there is a widening of the area suitable for *L. abortivum*, accompanied by a shift towards higher altitudes for both the 2030s and 2050s. In the SSP585 scenario, there is a habitat reduction at lower altitudes in both the 2030s and 2050s that is suitable for *L. abortivum*, with a shift towards higher altitude areas. In contrast to other myxotrophic species, which appear to be less susceptible to global warming, the reduction of the suitable climatic niche for this species was also identified in other studies [100,101]. In *C. rubra* and *E. microphylla*, it is observed that the greatest stability of area occupied by suitable habitats occurs in SSP245 2050s and SSP585 2030s, while for the remaining scenarios, the area suitable for the species is intensely reduced. The expansion of the distribution range of *L. abortivum*, as observed in other Mediterranean orchid species (e.g., *Orchis anthropophora* (L.) All., *O. purpurea* Huds. and *O. simia* Lam.), has been documented in conjunction with the contraction of the distribution range of *C. rubra* and *E. microphylla*, which are continental species (e.g., *Orchis militaris* L.) [76]. In contrast, studies have demonstrated that forest orchids do not experience a significant shift in their elevational distribution due to climate change [61,94]. The results of the present study provide partial confirmation of this hypothesis. In the continental species *C. rubra* and *E. microphylla*, there is a progressive reduction in suitable areas, rather than a shift towards higher altitudes, as is the case in *L. abortivum*. According to Jakubska-Busse et al. [102], the disappearance of occurrence sites of the genus *Cephalanthera* (including *C. rubra*) along the altitudinal gradient in Lower Silesia (SW Poland) is not due to recent climatic changes, but rather to the change in land use at the beginning of the 20th century. Similarly, for European *Epipactis* (including *E. microphylla*), environmental variables such as bedrock and land cover type appear to be more important than climatic variables alone in the prediction of distribution [103]. 

Habitat Suitability Models (HSMs) represent a valuable tool for species conservation, offering the potential to inform the planning of habitat and species protection actions [104,105]. Although they are typically employed to model the distribution of species at a large scale [106], the utilisation of HSMs for the conservation of threatened species is also possible at a fine scale [107], for instance, within protected areas (PAs). In the specific case of this study, the application of habitat suitability modelling in a PA in the Northern Apennines has demonstrated that fine-scale application can yield useful results for the conservation of plant species. The initial outcome demonstrates the suitability of ridge habitats for orchids in mesophilic environments (*C. rubra* and *E. microphylla*), which are already partially occupied by these species under the prevailing climatic conditions. Given the climate scenarios identified in the different cases, it is imperative that these areas are given the highest level of protection within the PA boundaries. This can be achieved through the establishment of new fully PAs or the modification of existing ones. The second result is similarly related to areas located at high altitudes, although in a different way. For thermophilic species, such as *L. abortivum*, these areas could serve as both expansion and refuge sites, where future conditions of suitability for the habitats to which the species are linked are envisaged. Consequently, these areas should be included in the planned conservation and protection plans for plant and animal species, and their protection should be a priority. The existing literature has demonstrated that climate change represents a significant threat to European conservation areas, necessitating the development of new protection policies [108]. In Italy, some studies have been conducted [35,109,110,111], and further analysis could be carried out in the future with the application of prioritisation tools (e.g., Marxan tool) within the boundaries of the PAs, to identify the areas that require protection in response to the new climate challenges [112,113].

## 4. Materials and Methods

### 4.1. Study Area

The Foreste Casentinesi, Monte Falterona and Campigna National Park (Figure 1) is a protected area (PA) situated in the Northern Apennines, specifically in the Tuscan-Romagna Apennines, on the border between the regions of Emilia-Romagna and Tuscany, straddling the provinces of Forlì-Cesena, Arezzo and Firenze. The PA encompasses an area of 36,400 hectares. One of the defining characteristics of the PA is the Apennine ridge, which runs north-west to south-east and divides the territory into two main slopes. The ridge’s highest altitudes are reached at the summits of Mount Falco (1657 m a.s.l.) and Mount Falterona (1654 m a.s.l.). The geology of the Romagna slope is characterised by sandstones and arenaceous marls (Middle-lower Miocene); in contrast, the Tuscan slope is dominated by sandstones and arenaceous marls (sometimes turbiditic) (Palaeogene) units [114]. A dividing feature between the two slopes is a belt of clays, limestones and clays (turbiditic) (Palaeogene) formations, which runs along the ridge in the central area [114]. Two additional areas of interest can be identified on the Tuscan slope. The first one is located at the southern border of the Park at La Verna, where skeletal limestones and calcarenites (Middle-lower Miocene) are present; the second one is situated between the localities of Moggiona and Camaldoli, where marls are found, occasionally containing pelagic facies chert (Middle-lower Miocene) [114]. The Park is situated in a temperate macrobioclimate and temperate oceanic bioclimate, exhibiting a weak Euroceanic subtype on the two sides, both the Tyrrhenian and the Adriatic; in contrast, the ridge area displays a semi-continental weak subtype [115]. The area can be divided into two different ombrotypes, namely a semi-arid lower horizon on the two sides and a semiarid upper horizon in the ridge area [115]. In terms of thermotype, three different classifications can be identified: supratemperate lower horizon for the lower areas of the two sides, supratemperate upper horizon for the central area that rises up to the ridge, and a third thermotype, orotemperate lower horizon, at the two highest peaks of the Park [115].

### 4.2. Species Occurrence Data

Target species occurrence data were obtained by the Foreste Casentinesi National Park Orchids Database [67]. Records are collected from 2000 to 2023; they include species occurrences from field research carried out in the PA territory, records collected by citizen science projects and data from bibliographic research [67]. Geographic coordinates are associated with each species occurrence collected in the field (datum: WGS84). The datasets were analysed by QGIS 3.32.3 software [116], R software [117] and RStudio [118]. To be used in the model, occurrence points were prepared through the following steps: duplicate records were removed first; then, records falling outside the boundary of the PA were identified and removed; finally, spatial thinning (or filtering) was applied using the R package *spThin* [119] to reduce spatial autocorrelation [120,121]. Although the redundancy of records is automatically reduced by MaxEnt algorithms which remove excess occurrence points leaving only one record for every single cell [81] the reduction of autocorrelation of spatial data from sampling error through specific methodologies can significantly improve the quality of ecological distribution models [122,123,124]. The distance used as the thinning parameter (thin.par) is 1 km, which is larger than the reference grid cell size (≃1 km). 

### 4.3. Environmental Variables

To develop the distribution models, the environmental variables that most influence the global distribution of plant species were considered [125]. Specifically, climatic, edaphic, topographic, anthropic and land cover variables were used. Bioclimatic variables (BIO1-BIO19) and elevation (ELV) were obtained from the WorldClim2.1 climate database [126]. The variables Slope (SLP) and Aspect (ASP) were extracted with the QGIS 3.32.3 software [116] starting from elevation data. In total, there are 3 topographic variables. The land cover variables (LC1-LC12) were obtained from the EarthEnv Global 1-km Consensus Land Cover database [127]. The anthropic impact variable (CHP) was obtained from the Last of the Wild Project, Version 3 (LWP-3): 2009 Human Footprint, 2018 Release database [128,129]. The edaphic variables (SOIL1-SOIL11) were obtained from the ISRIC SoilGrids250m 2.0 database and are referred to a depth of 0–5 cm of soil [130,131]. In total 46 ecological variables were used, at a spatial resolution of 30 arc-seconds (usually referred to as ‘1-km’ spatial resolution) which corresponds to a surface of ≃ 0.86 km^2^ at the equator [126].

One of the main problems of species distribution models is the choice of environmental variables to include in the model. This is because collinearity exists between environmental variables and this can very negatively influence the quality of the model in various ways [132,133]. As a result, it is essential to test multicollinearity between environmental variables with appropriate statistical analyses to avoid this [134,135]. In this work, the analyses were carried out using the software R [117], R studio [118] and Past 4.04 [136]. The workflow used to identify the best environmental variables among those identified was taken up and integrated based on other studies [120,137] and consists of several sequential steps listed below:

1. Data matrixes creation: using the QGIS 3.32.3 software [116] we combined each species occurrence record with the spatial value of the corresponding attribute for the 46 variables, i.e., the cell value of the environmental variable in which the occurrence point falls. A matrix for all investigated species was obtained.
2. EV screening and ranking: A preliminary modelling exercise was initiated utilising MaxEnt to identify the number and nature of environmental variables influencing the model. The initial model was constructed by applying the default ‘Auto features’ setting (default FC and RM settings) and then three replicate runs were initiated. The mean value of permutation importance for each of the variables included in the model in the three runs was then obtained [138]. The jackknife test [139] was applied to assess the significance of each environmental variable in elucidating the distribution of a species within the MaxEnt model. This enables the assessment of the impact of predictors on the model performance in terms of gain. Variables that lead to a reduction in gain when excluded are considered more important during the modelling process. The light blue bars indicate the impact on the model when the single variable is not included, the dark blue bars indicate the impact with only the variable included, and the red bar indicates the inclusion of all variables. Following, a ranking of the variables was established according to their permutation importance in the model. The use of permutation importance over percent contribution is preferable because it depends on the final model, not on the path used for each run, and this is better for correctly assessing the importance of each variable [140,141]. Furthermore, we decided to directly eliminate variables with a small contribution rate to the model, as this was deemed to be too low [141,142,143]. Variables with a contribution rate of less than 1% were removed [144,145].
3. Multicollinearity test #1: Pearson correlation coefficient (r) between each pair of environmental variables was calculated using Past 4.04 software [136]. If |r| ≥ 0.8 [135,145,146,147,148] there is a correlation between the variables and one of the two must be excluded. Based on the permutation importance rank, in the screening and ranking phase, the variable with the greatest contribution for the model was retained while the second was discarded. This operation was performed for each pair of environmental variables.
4. Multicollinearity test #2: to further reduce the multicollinearity between the environmental variables selected by the first multicollinearity test, Variance Inflation Factor (VIF), was calculated with the R package *usdm* [149]. A precautionary threshold was chosen at 5. Variables with VIF > 5 were excluded because they were strongly correlated with each other [84,135,150,151].
5. Final environmental dataset: finally, variables that respect both multicollinearity conditions tests (|r| ≤ 0.8 and VIF < 5) were chosen as final predictors. From these, based on the ranking obtained in the screening and ranking phase, the top five variables contributing to the model were selected, sorted according to permutation importance values. These variables, five for each species, are used to build the final models.

In addition to investigating habitat suitability for current climatic conditions, the study focuses on identifying the most suitable habitats for changing climatic conditions in the near future. To do this, we used bioclimatic data (BIO1-BIO19) referring to the future, i.e., 2030s (average values from 2021 to 2040) and 2050s (average values from 2041 to 2060). These data, as for the current conditions (1970–2000), were obtained from the WorldClim2.1 climate database [126]. For each future period, we selected two Shared Socioeconomic Pathways (SSPs), SSP2-4.5 “Middle of the road”, and SSP5-8.5 “Taking the Highway” developed within the Panel on Climate Change (IPCC) and used in the Coupled Model Intercomparison Project Phase 6 (CMIP6) to estimate future climate change [152]. The Global Climate Model (GCM) from which the scenarios were selected is the CMCC-ESM2 of the Euro-Mediterranean Center on Climate Change [153,154]. Each Shared Socio-economic Pathway [155] is associated with a narrative [156,157] that describes socioeconomic change for the future with demographic, economic, energy, land use and greenhouse gas and air pollutant emissions drivers, developed by models [158,159,160]. The SSP2-4.5 estimate is based on intermediate greenhouse gas (GHG) emissions with CO_2_ emissions around current levels until 2050, after which they are projected to fall but not reach net zero by 2100 [152,161]. Conversely, SSP5-8.5 predict very high GHG emissions with CO_2_ emissions tripling by 2075 [152,161]. The estimated warming for the 2041–2060 and 2081–2100 periods in these scenarios is 2.0 °C and 2.7 °C for the SSP2-4.5 scenario and 2.4 °C and 4.4 °C for SSP5-8.5, respectively, with a likely range in 2081–2100 of 2.1 °C–3.5 °C for SSP2 4.5 and 3.3 °C–5.7 °C for SSP5 8.5 [152,162]. This paper assumes that all other variables in the MaxEnt model, except the 19 climate variables, will not change in future years [137].

### 4.4. Final Model Construction, Optimization and Evaluation

MaxEnt software version 3.4.4 [163,164] was used to predict the habitat suitability of the three species reported in this work. In the models, 75% of the occurrence data was randomly selected and used as a model training set, while the remaining 25% was used as a model validation set. We have set the modelling parameters in the settings panel as follows: maximum number of iterations set to 500, ‘Bootstrap’ replication method and 10 model replications. Furthermore, the response curve and jackknife test parameters have been enabled to analyse the effects and contribution of each environmental variable. We left the convergence threshold to the default value (1 × 10^−5^). The output format chosen is cloglog (complementary log-log). The use of default parameters in MaxEnt models, although applied in many studies, can lead to overfitting problems and the production of over-complex or over-simplistic models [165,166,167]. To avoid these problems, we used the R package *ENMeval* [168,169] to adjust two parameters of the MaxEnt model, namely regularization multipliers (RM) and feature combinations (FC). By default, these parameters are set by MaxEnt as RM = 1 and as FC = LQHPT where L = linear, Q = quadratic, H = hinge, P = product and T = threshold. These parameters can be adjusted to achieve a more refined model [81,170] and this has been done through the use of ENMeval metrics. The best set of combinations of the RM and FC parameters was identified based on the Akaike information criterion correction (AICc). The lowest value of the AICc delta (ΔAICc = 0) calculated by *ENMeval* identifies better parameters value [82,145,171] and corresponds to the best model in terms of goodness-of-fit and complexity [168,172]. The degree of overfitting of the model can be effectively quantified through the comparison of two other ENMeval metrics: the difference between the AUC training and AUC testing value (AUC _DIFF_ = AUC _TRAIN_ − AUC _TEST_) and the 10% training omission rate (OR _10_) [82,83,168]. High values of AUC _DIFF_ and OR _10_ > 0.1 indicate overfitting of the models [80,81]. Finally, the accuracy of the model was evaluated using the Receiver Operating Characteristic (ROC) curves and the area under the ROC curve or AUC values (Area Under Curve), a method widely used in predicting habitat suitability using the MaxEnt algorithm [173,174]. The AUC value has a range from 0.5 to 1, where 0.5 indicates that the model does not predict better than a random distribution. The higher the value, the better the model prediction and the greater the correlation between the presence of the species and the environmental characteristics. Based on the AUC value, the forecast is classified as fail or inadequate (0.5–0.6), poor (0.6–0.7), reasonable or fair (0.7–0.8), good (0.8–0.9), perfect or excellent (0.9–1.0) [82,83,84].

### 4.5. Habitat Suitability Analysis and Visualization

MaxEnt model chosen for each of the analysed species, under current and future conditions were imported into QGIS [116], R [117] and Rstudio [118]. MaxEnt generates cartography where each cell is associated with a suitability index that ranges from 0 to 1. For low values, there are minimal probabilities of finding suitable habitats, while for high values, the probabilities increase [40,164]. In order to effectively discriminate the areas classifiable as unsuitable and suitable, the average value of the Maximum Test Sensitivity Plus Specificity (MTSPS) was applied as a threshold [120,175,176,177]. It has been demonstrated that this threshold leads to effective results in models based on presence data only [178,179,180]. The MaxEnt outputs were therefore reclassified based on the probability (*p*) of habitat suitability occurrence. For probability values below the MTSPS threshold (*p* < MTSPS), the output was classified as unsuitable for the species. Conversely, for probability values above the MTSPS threshold (*p* > MTSPS), the output was further classified into three suitability classes at equal size intervals [144,181,182]: low suitability, moderate suitability and high suitability [120,147,171]. Furthermore, the outputs were reclassified using the presence/absence (1/0) format of suitable habitats using the MTSPS threshold. With the R package *biomod2* [183,184] the range change between current and future models was calculated [171,185].

## Figures and Tables

**Table 1:** The environmental variables (EVs) involved in the process of predicting habitat suitability. Only the variables highlighted in bold were included in the final MaxEnt model and actively participated in the modelling process.

ID	Code	Variable Description	Unit
1	BIO1	Annual Mean Temperature	°C
2	BIO2	Mean Diurnal Range (Mean of monthly (max temp—min temp))	°C
3	BIO3	Isothermality (BIO2/BIO7) (×100)	%
4	**BIO4**	**Temperature Seasonality (standard deviation ×100)** ^3^	**%**
5	BIO5	Max Temperature of Warmest Month	°C
6	BIO6	Min Temperature of Coldest Month	°C
7	BIO7	Temperature Annual Range (BIO5-BIO6)	°C
8	BIO8	Mean Temperature of Wettest Quarter	°C
9	BIO9	Mean Temperature of Driest Quarter	°C
10	BIO10	Mean Temperature of Warmest Quarter	°C
11	**BIO11**	**Mean Temperature of Coldest Quarter** ^3^	**°C**
12	BIO12	Annual Precipitation	mm
13	BIO13	Precipitation of Wettest Month	mm
14	**BIO14**	**Precipitation of Driest Month** ^2,3^	**mm**
15	**BIO15**	**Precipitation Seasonality (Coefficient of Variation)** ^1,2^	**%**
16	BIO16	Precipitation of Wettest Quarter	mm
17	BIO17	Precipitation of Driest Quarter	mm
18	**BIO18**	**Precipitation of Warmest Quarter** ^1^	**mm**
19	BIO19	Precipitation of Coldest Quarter	mm
20	LC01	Evergreen/Deciduous Needleleaf Trees	%
21	LC02	Evergreen Broadleaf Trees	%
22	LC03	Deciduous Broadleaf Trees	%
23	LC04	Mixed/Other Trees	%
24	LC05	Shrubs	%
25	LC06	Herbaceous Vegetation	%
26	LC07	Cultivated and Managed Vegetation	%
27	LC08	Regularly Flooded Vegetation	%
28	LC09	Urban/Built-up	%
29	LC10	Snow/Ice	%
30	**LC11**	**Barren** ^3^	**%**
31	LC12	Open Water	%
32	**SOIL1**	**Bulk density of the fine earth fraction** ^1,2,3^	**cg/cm^3^**
33	SOIL2	Cation Exchange Capacity of the soil	mmol(c)/kg
34	**SOIL3**	**Volumetric fraction of coarse fragments (>2 mm)** ^1^	**cm^3^/dm^3^ (vol‰)**
35	**SOIL4**	**Proportion of clay particles (<0.002 mm) in the fine earth fraction** ^2^	**g/kg**
36	SOIL5	Total nitrogen (N)	cg/kg
37	SOIL6	Soil pH	pHx10
38	SOIL7	Proportion of sand particles (>0.05 mm) in the fine earth fraction	g/kg
39	SOIL8	Proportion of silt particles (≥0.002 mm and ≤0.05 mm) in the fine earth fraction	g/kg
40	SOIL9	Soil organic carbon content in the fine earth fraction	dg/kg
41	SOIL10	Organic carbon density	hg/m^3^
42	SOIL11	Organic carbon stocks	t/ha
43	**SLP**	**Slope** ^2^	**degree**
44	ELV	Elevation	meter
45	ASP	Aspect	degree
46	**CHP**	**Cumulative human pressure on the environment** ^1^	

(^1^) Variable used in the *C. rubra* model; (^2^) Variable used in the *E. microphylla* model; (^3^) Variable used in the *L. abortivum* model.

**Table 2:** Permutation importance and percent contribution values obtained from the modelling for each species.

Species	Environmental Variables (EV)	Permutation Importance (%)	Percent Contribution (%)
*Cephalanthera rubra*	SOIL1 BIO18 BIO15 CHP SOIL3	35.5 25.2 18.5 11.4 9.5	34 24.7 8.1 27.4 5.8
*Epipactis microphylla*	SOIL1 BIO14 SOIL4 SLOPE BIO15	46.6 19 17.6 12.5 10.4	45 18.3 21.4 8.6 6.7
*Limodorum abortivum*	BIO04 BIO14 SOIL1 BIO11 LC11	39.5 35.2 17.7 6.7 1	30.2 32.5 28.9 7.4 1

**Table 3:** Classification of habitat suitability based on probability values obtained from the final MaxEnt model of *C. rubra*. The area is expressed in square kilometres (km^2^), with the percentage (%) referring to the total area studied. The percentage increase (% inc.) or decrease (% dec.) is expressed in positive (+) or negative (−) values in comparison to the total area currently occupied.

Time	Scenario	Unit	Tot.	Unsuitability	Low Suitability	Moderate Suitability	High Suitability
Present		km^2^	315.28	97.92	144.44	113.89	56.94
		%	76.30	23.70	34.96	27.56	13.78
2030s	SSP245	km^2^	140.28	272.92	85.42	46.53	8.33
		%	33.95	66.05	20.67	11.26	2.02
		% inc./dec.	−55.51	+178.72	−40.87	−59.15	−85.37
	SSP585	km^2^	294.44	118.75	166.67	81.25	46.53
		%	71.26	28.74	40.34	19.66	11.26
		% inc./dec.	−6.61	+21.28	+15.38	−28.66	−18.29
2050s	SSP245	km^2^	281.94	131.25	166.67	80.56	34.72
		%	68.24	31.76	40.34	19.50	8.40
		% inc./dec.	−10.57	+34.04	+15.38	−29.27	−39.02
	SSP585	km^2^	27.08	386.11	25.69	1.39	0.00
		%	6.55	93.45	6.22	0.34	0.00
		% inc./dec.	−91.41	+294.33	−82.21	−98.78	−100.00

**Table 4:** Classification of habitat suitability based on probability values obtained from the final MaxEnt model of *E. microphylla*. The area is expressed in square kilometres (km^2^), with the percentage (%) referring to the total area studied. The percentage increase (% inc.) or decrease (% dec.) is expressed in positive (+) or negative (−) values in comparison to the total area currently occupied.

Time	Scenario	Unit	Tot.	Unsuitability	Low Suitability	Moderate Suitability	High Suitability
Present		km^2^	225.69	187.50	107.64	79.17	38.89
		%	54.62	45.38	26.05	19.16	9.41
2030s	SSP245	km^2^	144.44	268.75	97.92	37.50	9.03
		%	34.96	65.04	23.70	9.08	2.18
		% inc./dec.	−36.00	+43.33	−9.03	−52.63	−76.79
	SSP585	km^2^	198.61	214.58	115.97	64.58	18.06
		%	48.07	51.93	28.07	15.63	4.37
		% inc./dec.	−12.00	+14.44	+7.74	−18.42	−53.57
2050s	SSP245	km^2^	195.14	218.06	120.83	51.39	22.92
		%	47.23	52.77	29.24	12.44	5.55
		% inc./dec.	−13.54	+16.30	+12.26	−35.09	−41.07
	SSP585	km^2^	11.81	401.39	11.81	0.00	0.00
		%	2.86	97.14	2.86	0.00	0.00
		% inc./dec.	−94.77	+114.07	−89.03	−100.00	−100.00

**Table 5:** Classification of habitat suitability based on probability values obtained from the final MaxEnt model of *L. abortivum*. The area is expressed in square kilometres (km^2^), with the percentage (%) referring to the total area studied. The percentage increase (% inc.) or decrease (% dec.) is expressed in positive (+) or negative (−) values in comparison to the total area currently occupied.

Time	Scenario	Unit	Tot.	Unsuitability	Low Suitability	Moderate Suitability	High Suitability
Present		km^2^	217.36	195.83	93.75	82.64	40.97
		%	52.61	47.39	22.69	20.00	9.92
2030s	SSP245	km^2^	320.83	92.36	59.72	95.14	165.97
		%	77.65	22.35	14.45	23.03	40.17
		% inc./dec.	+47.60	−52.84	−36.30	+15.13	+305.08
	SSP585	km^2^	202.78	210.42	91.67	90.97	20.14
		%	49.08	50.92	22.18	22.02	4.87
		% inc./dec.	−6.71	+7.45	−2.22	+10.08	−50.85
2050s	SSP245	km^2^	309.03	104.17	75.69	100.69	132.64
		%	74.79	25.21	18.32	24.37	32.10
		% inc./dec.	+42.17	−46.81	−19.26	+21.85	+223.73
	SSP585	km^2^	79.17	334.03	70.83	8.33	0.00
		%	19.16	80.84	17.14	2.02	0.00
		% inc./dec.	−63.58	+70.57	−24.44	−89.92	−100.00

**Table 6:** Distribution area changes in *C. rubra* for different climate scenarios at two different periods.

Time	Scenario	Units	CRS	Loss	Stable	Gain	%Loss	%Gain	SRC
Present		cells	454						
		km^2^	315.28						
2030s	SSP245	cells		252	202	0	55.51	0.00	−55.51
		km^2^		175.00	140.28	0.00			
	SSP585	cells		34	420	4	7.49	0.88	−6.61
		km^2^		23.61	291.67	2.78			
2050s	SSP245	cells		49	405	1	10.79	0.22	−10.57
		km^2^		34.03	281.25	0.69			
	SSP585	cells		415	39	0	91.41	0.00	−91.41
		km^2^		288.19	27.08	0.00			

**Table 7:** Distribution area changes in *E. microphylla* for different climate scenarios at two different periods.

Time	Scenario	Units	CRS	Loss	Stable	Gain	% Loss	% Gain	SRC
Present		cells	325						
		km^2^	225.69						
2030s	SSP245	cells		117	208	0	36.00	0.00	−36.00
		km^2^		81.25	144.44	0.00			
	SSP585	cells		39	286	0	12.00	0.00	−12.00
		km^2^		27.08	198.61	0.00			
2050s	SSP245	cells		44	281	0	13.54	0.00	−13.54
		km^2^		30.56	195.14	0.00			
	SSP585	cells		308	17	0	94.77	0.00	−94.77
		km^2^		213.89	11.81	0.00			

**Table 8:** Distribution area changes in *L. abortivum* for different climate scenarios at two different periods.

Time	Scenario	Units	CRS	Loss	Stable	Gain	% Loss	% Gain	SRC
Present		cells	313						
		km^2^	217.36						
2030s	SSP245	cells		60	253	209	19.17	66.77	47.60
		km^2^		41.67	175.69	145.14			
	SSP585	cells		152	161	131	48.56	41.85	−6.71
		km^2^		105.56	111.81	90.97			
2050s	SSP245	cells		64	249	196	20.45	62.62	42.17
		km^2^		44.44	172.92	136.11			
	SSP585	cells		297	16	98	94.89	31.31	−63.58
		km^2^		206.25	11.11	68.06			

## Data Availability

All data necessary to replicate this study’s results are included in this published article (and its Supplementary Materials). Raw data are available upon request.

## Supplementary Materials

The following supporting information can be downloaded at: https://www.mdpi.com/article/10.3390/plants13131810/s1, Figure S1: Pearson’s correlation table of *C. rubra*; Figure S2: Pearson’s correlation table of *E. microphylla*; Figure S3: Pearson’s correlation table of *L. abortivum*; Figure S4: Response curve of environmental factors of *C. rubra*; Figure S5: Response curve of environmental factors of *E. microphylla*; Figure S6: Response curve of environmental factors of *L. abortivum*; Figure S7: Goodness-of-fit test of the distribution model created for *C. rubra*; Figure S8: Goodness-of-fit test of the distribution model created for *E. microphylla*; Figure S9: Goodness-of-fit test of the distribution model created for *L. abortivum*; Table S1: Reduced occurence points of *C. rubra*, *E. microphylla* and *L. abortivum*; Table S2: Variance Inflation Factors.
