# Supplementary material for: Forest Orchids under Future Climate Scenarios: Habitat Suitability Modelling to Inform Conservation Strategies
Source: Plants (Basel). 2024 Jun 30;13(13):1810. doi: 10.3390/plants13131810 (PMC11243989; doi:10.3390/plants13131810)
Supplement: Supplementary file 1 [file plants-13-01810-s001.zip › plants-3062157-supplementary.pdf]

	Cephalanthera rubra		*Epipactis microphylla*		*Limodorum abortivum*	
**ID**	**Long**	**Lat**	**Long**	**Lat**	**Long**	**Lat**
**1**	11,708245	43,891257	11,708245	43,891257	11,795190	43,925821
**2**	11,747015	43,958646	11,687583	43,927517	11,755750	43,961867
**3**	11,721036	43,964506	11,932549	43,710116	11,926903	43,746414
**4**	11,724476	43,977175	11,797805	43,879971	11,691217	43,928833
**5**	11,735129	43,966953	11,733523	43,943719	11,757908	43,899523
**6**	11,672503	44,012987	11,726421	43,935071	11,782292	43,938181
**7**	11,698705	43,958796	11,798249	43,852197	11,837736	43,836313
**8**	11,734158	43,993957	11,701975	43,961639	11,769201	43,944752
**9**	11,790129	43,957477	11,659354	43,963103	11,739502	43,963928
**10**	11,728818	43,891687	11,793537	43,870236	11,830902	43,850867
**11**	11,712980	44,023697	11,947150	43,720962	11,671432	43,967220
**12**	11,894184	43,793517	11,895576	43,775788	11,659354	43,963103
**13**	11,778444	43,849949	11,817802	43,944259	11,646492	43,975745
**14**	11,792455	43,844193	11,766917	43,852401	11,770328	43,881569
**15**	11,817958	43,832035	11,700175	43,880697	11,795925	43,885671
**16**	11,700737	44,014178	11,743009	43,855058	11,915014	43,822815
**17**	11,740448	43,944024	11,730194	43,870261	11,707978	43,943418
**18**	11,700454	43,935107	11,914269	43,798465	11,796876	43,782154
**19**	11,745160	43,929236	11,744017	43,872968	11,809918	43,781624
**20**	11,723726	43,950008	11,813887	43,783705	11,806134	43,761452
**21**	11,722180	43,940710	11,822124	43,809628	11,745853	43,951137
**22**	11,879272	43,799553			11,895902	43,830788
**23**	11,874381	43,791283			11,756317	43,929869
**24**	11,857986	43,773533			11,864007	43,874880
**25**	11,790587	43,792330			11,761446	43,985699
**26**	11,836357	43,882975			11,708834	44,013064
**27**	11,807958	43,881555			11,666022	44,005749
**28**	11,760176	43,926697			11,725375	43,996809
**29**	11,738715	43,916607			11,774878	43,892883
**30**	11,804132	43,922076			11,819183	43,927148
**31**	11,802380	43,934235			11,720545	43,938856
**32**	11,941453	43,714527			11,706727	44,029894
**33**	11,700364	43,922457			11,836357	43,882975
**34**	11,932537	43,707686			11,807958	43,881555
**35**	11,683389	43,931597			11,769354	43,922946
**36**	11,744017	43,872968			11,738715	43,916607
**37**	11,813887	43,783705			11,700364	43,922457
**38**					11,697832	43,953852
**39**					11,938167	43,701045

Table S1: Reduced occurence points of *C. rubra, E. microphylla* and *L. abortivum*

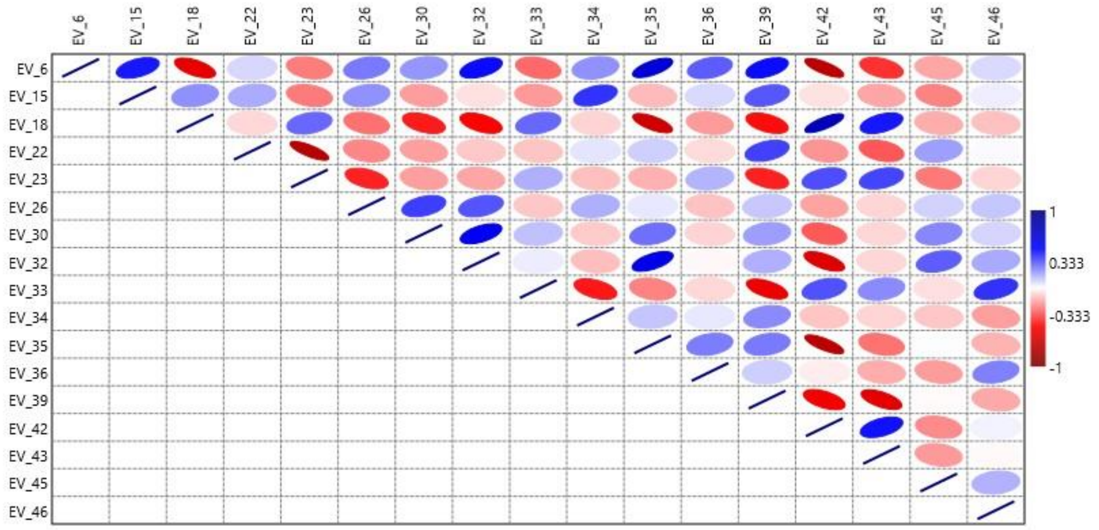

Figure S1: Pearson's correlation table of *C. rubra*

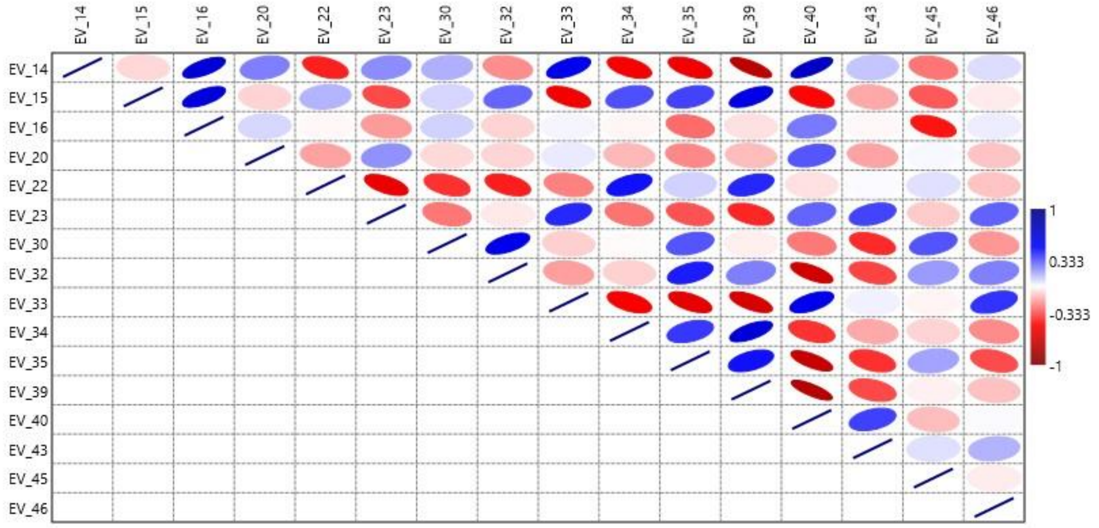

Figure S2: Pearson's correlation table of *E. microphylla*

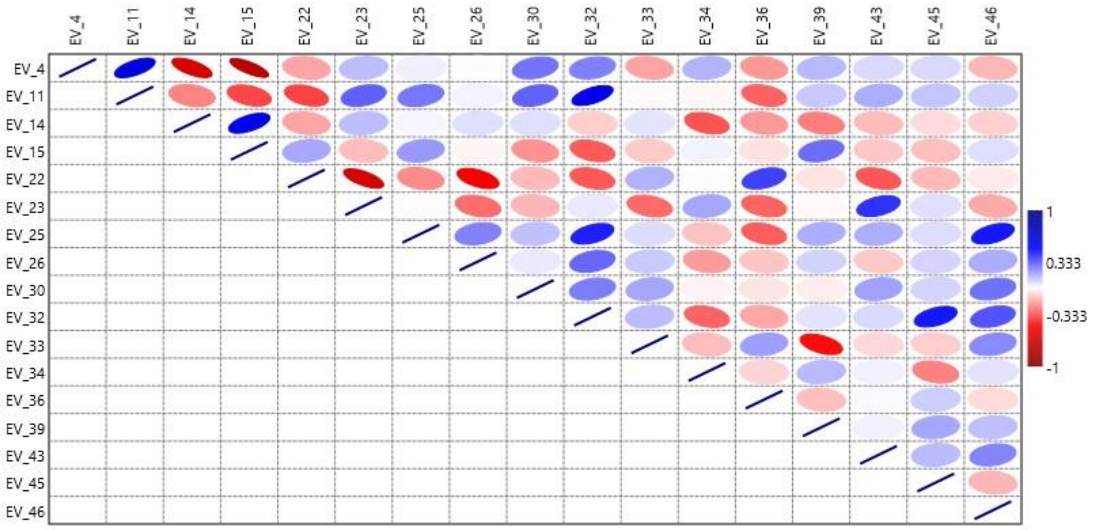

Figure S3: Pearson's correlation table of *L. abortivum*

Cephalanthera rubra		*Epipactis microphylla*		*Limodorum abortivum*	
**EVs**	**VIF**	**EVs**	**VIF**	**EVs**	**VIF**
BIO15	2,3	BIO14	2,94	BIO4	4,63
BIO18	4,78	BIO15	1,74	BIO11	2,91
LC04	1,62	LC03	2,52	BIO14	4,77
LC07	2,05	SOIL1	2,84	LC03	3,21
LC11	1,99	SOIL3	1,95	LC06	1,53
SOIL1	2,53	SOIL4	2,12	LC07	2,19
SOIL2	2,44	SLP	1,41	LC11	1,54
SOIL3	1,61			SOIL1	2,95
SOIL4	3,4			SOIL3	1,74
SOIL8	3,72			SOIL5	1,79
SLP	1,9			SOIL8	1,23
CHP	1,74			SLP	1,77
				ASP	1,55

Table S2: Variance Inflation Factors

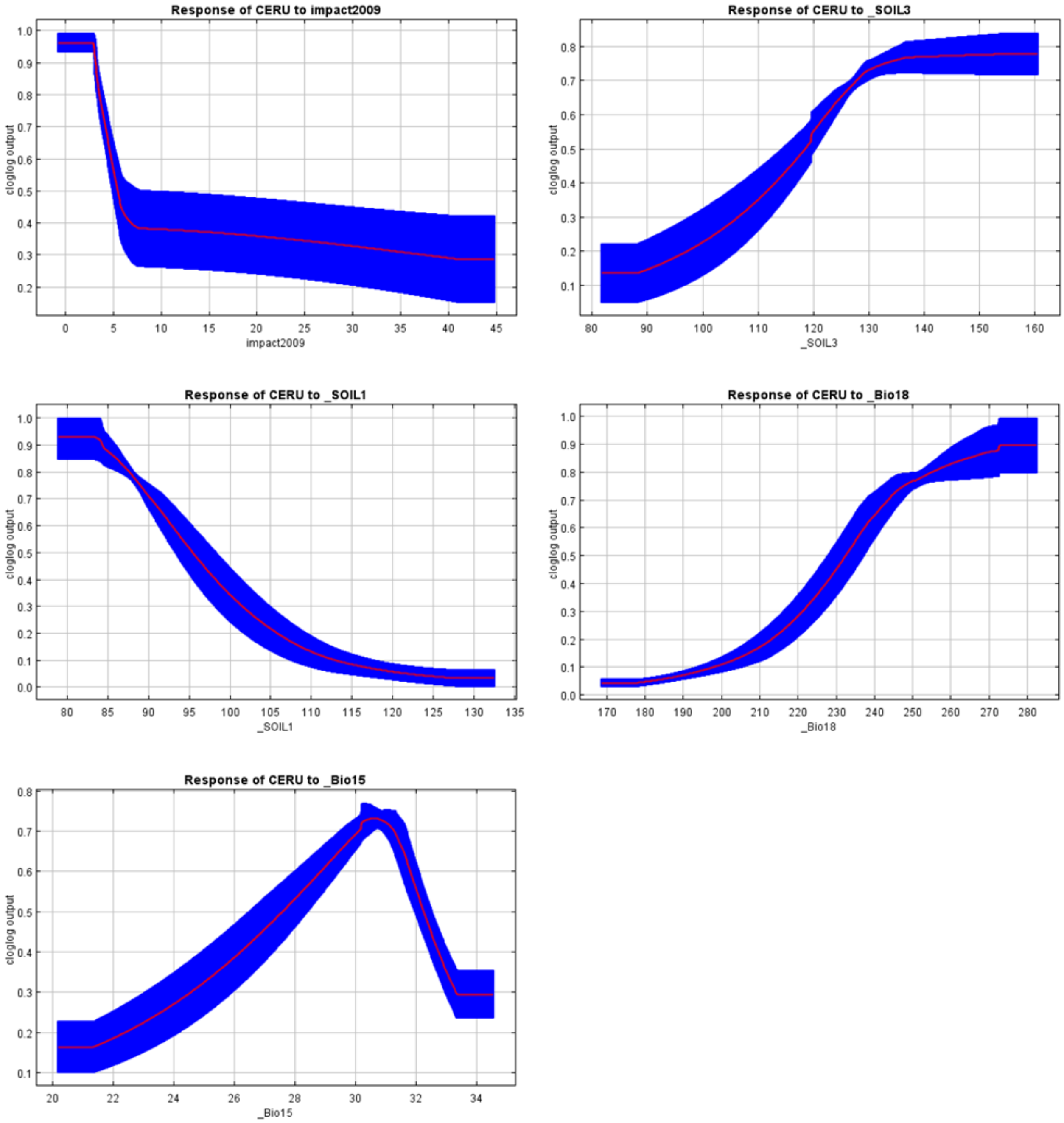

Figure S4: Response curve of environmental factors of *C. rubra*

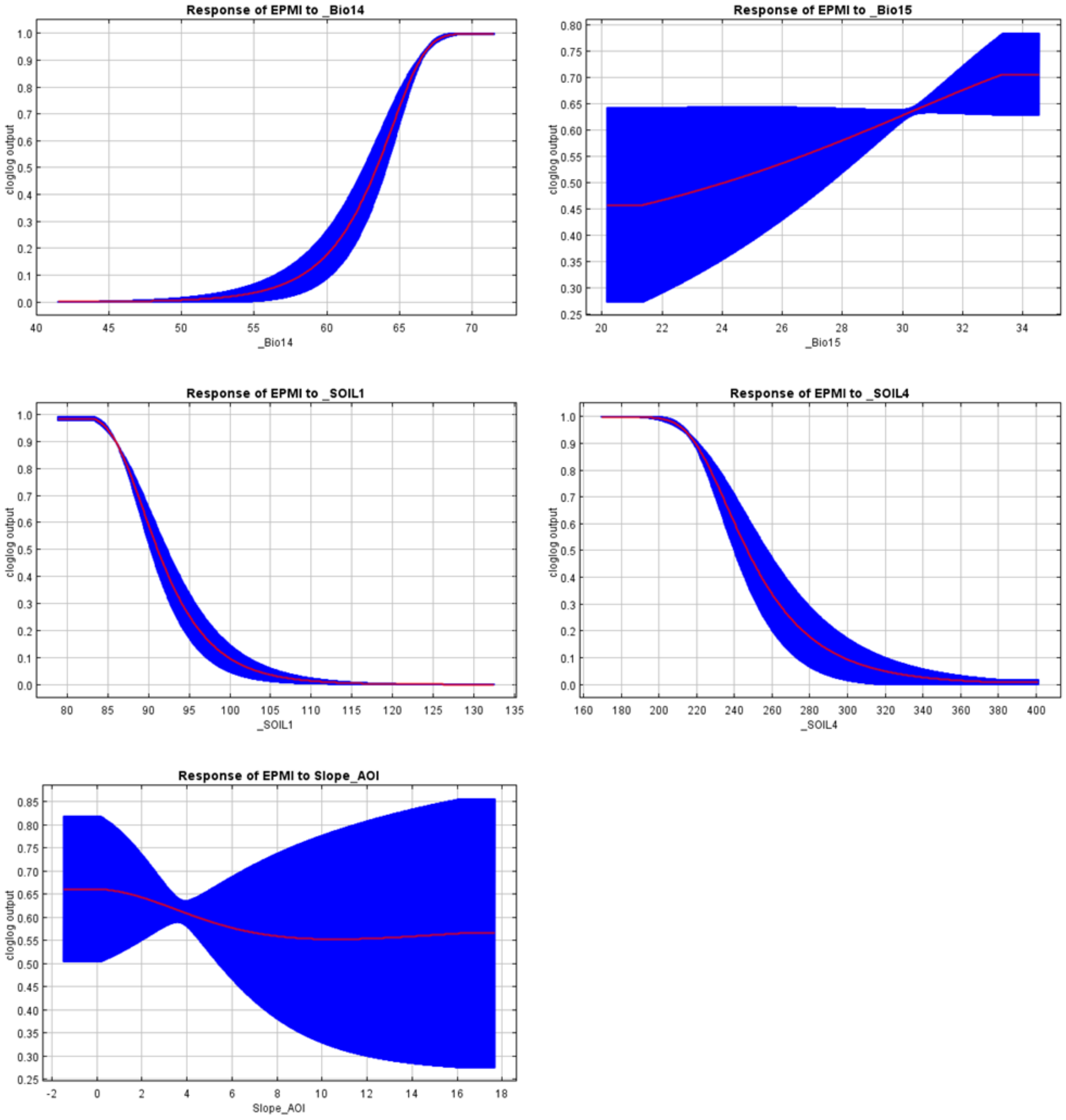

Figure S5: Response curve of environmental factors of *E. microphylla*

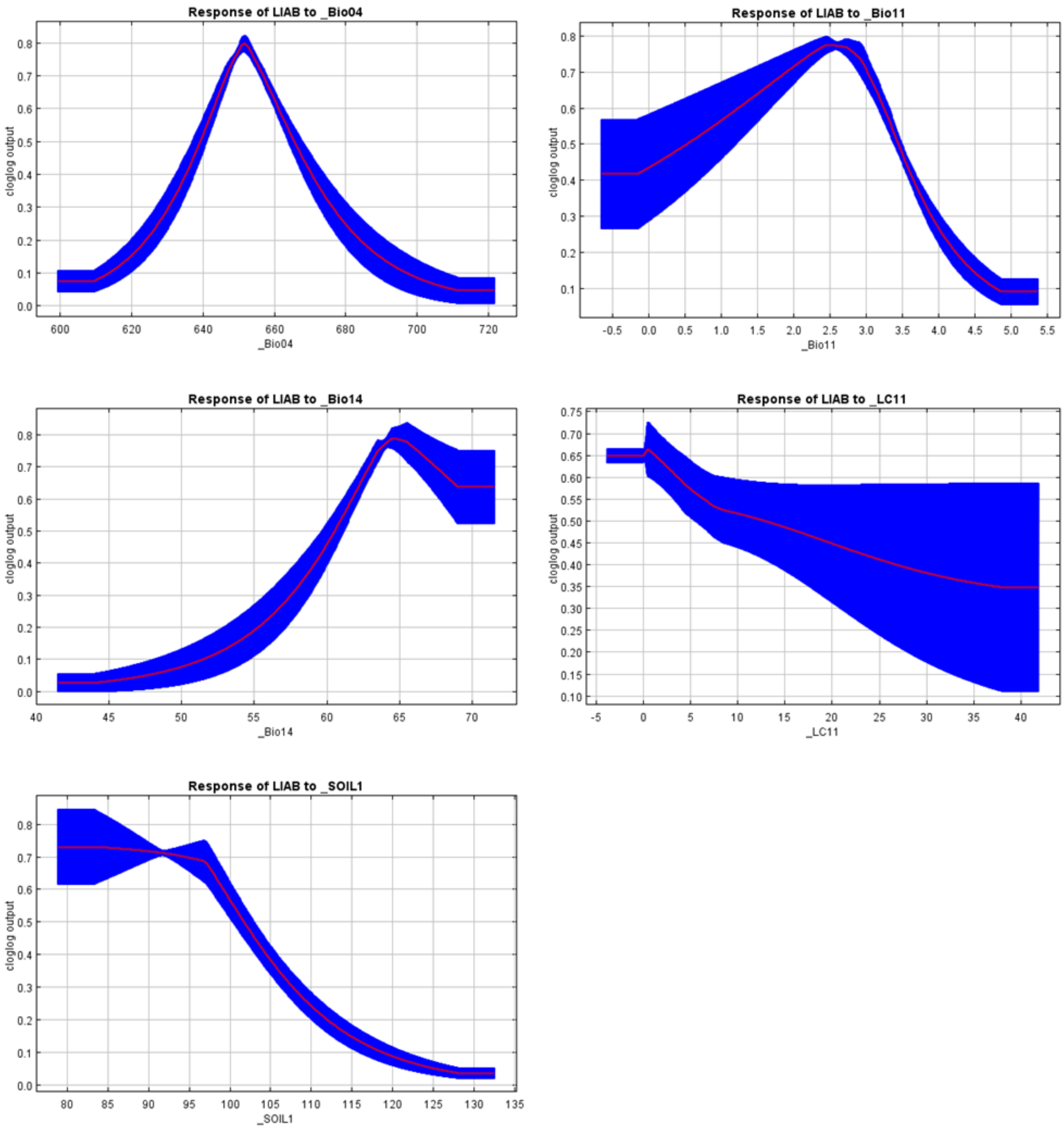

Figure S6: Response curve of environmental factors of *L. abortivum*

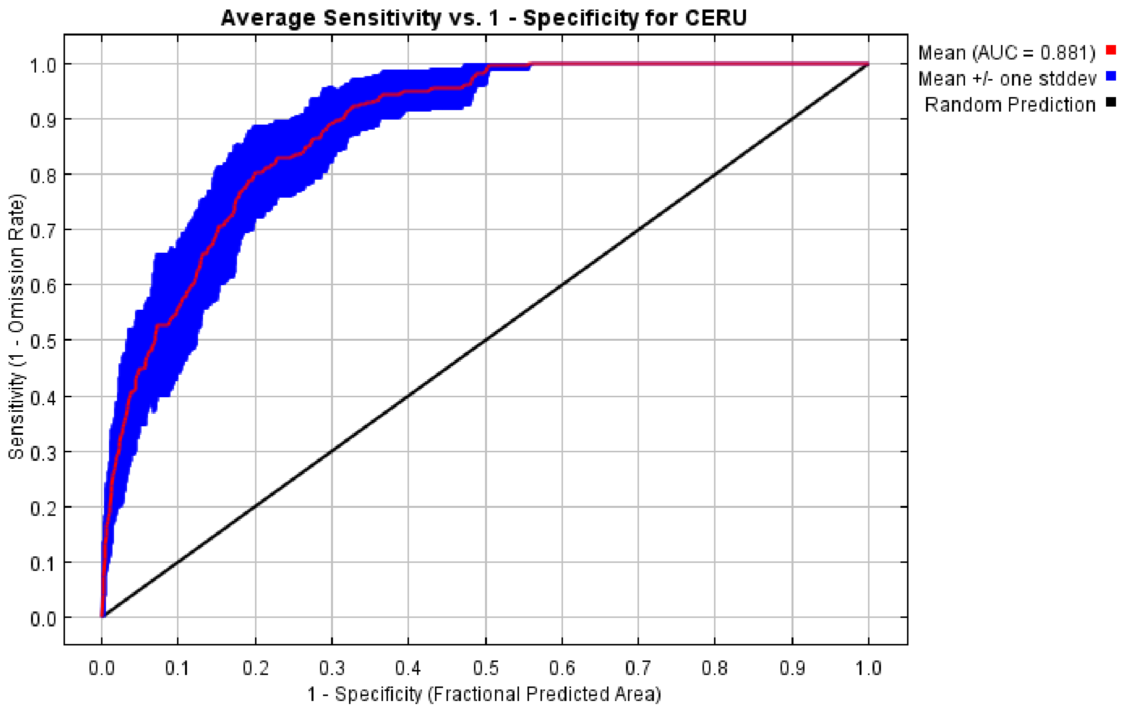

Figure S7: Goodness-of-fit test of the distribution model created for *C. rubra*

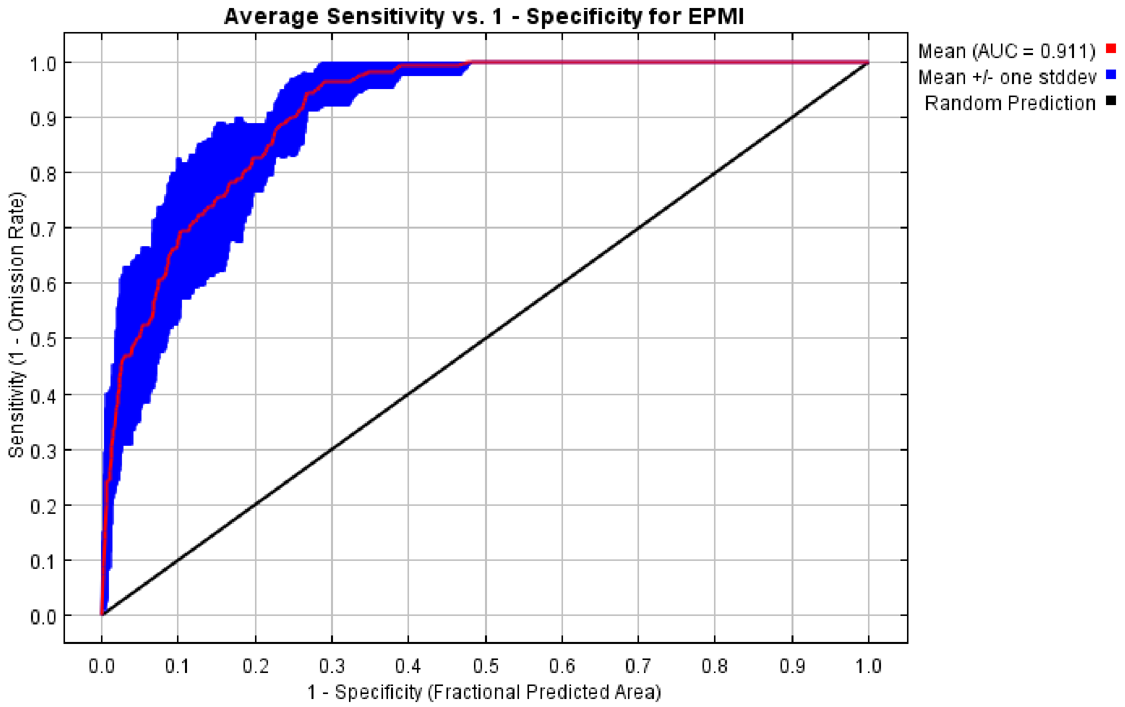

Figure S8: Goodness-of-fit test of the distribution model created for *E. microphylla*

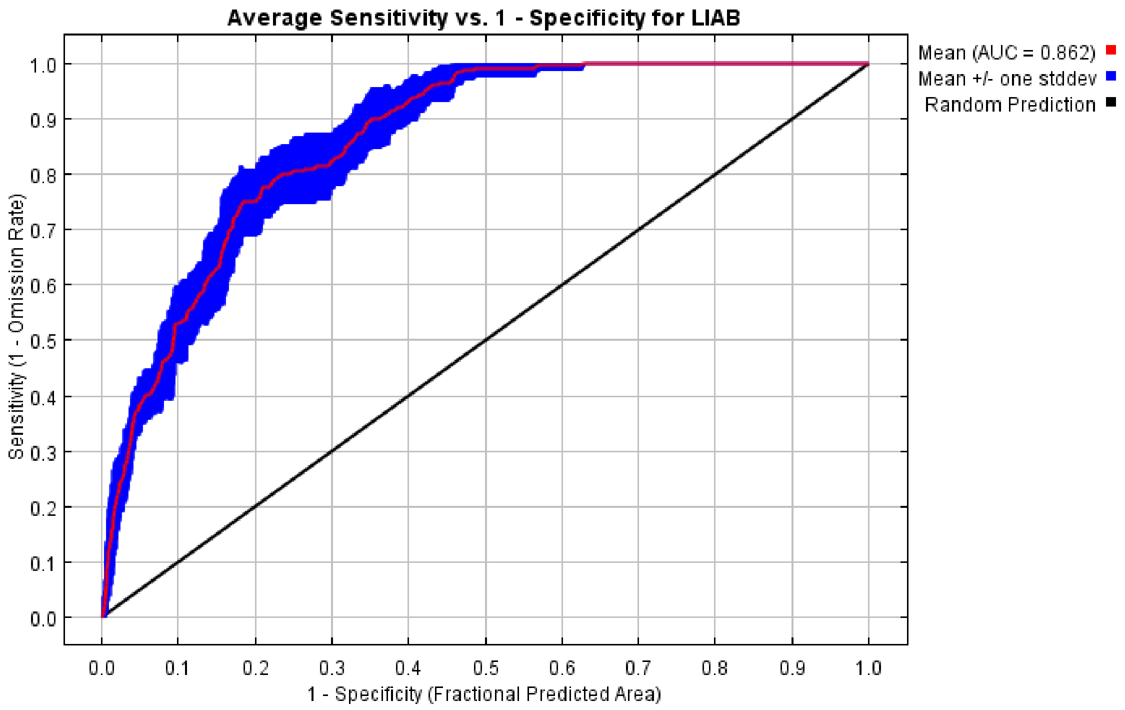

Figure S9: Goodness-of-fit test of the distribution model created for *L. abortivum*
